# Influence of Aza-Glycine Substitution on the Internalization of Penetratin

**DOI:** 10.3390/pharmaceutics16040477

**Published:** 2024-03-30

**Authors:** Karima Tarchoun, Dóra Soltész, Viktor Farkas, Ho-Jin Lee, Ildikó Szabó, Zoltán Bánóczi

**Affiliations:** 1 Institute of Chemistry, Faculty of Science, ELTE Eötvös Loránd University, Pázmány Péter sétány 1/A, 1117 Budapest, Hungary; tarchoun.karima14@gmail.com (K.T.); soltesz.dora6@gmail.com (D.S.); 2 Hevesy György PhD School of Chemistry, Institute of Chemistry, ELTE Eötvös Loránd University, Pázmány Péter sétány 1/A, 1117 Budapest, Hungary; 3 HUN-REN-ELTE Protein Modeling Research Group, Institute of Chemistry, ELTE Eötvös Loránd University, Pázmány Péter sétány 1/A, 1117 Budapest, Hungary; farkas.viktor@ttk.elte.hu; 4 Department of Natural Sciences, Southwest Tennessee Community College, Memphis, TN 38015, USA; hlee3@southwest.tn.edu; 5 Division of Natural and Mathematics Sciences, LeMoyne-Own College, Memphis, TN 38126, USA; 6 HUN-REN-ELTE Research Group of Peptide Chemistry, 1117 Budapest, Hungary; szaboi8@gmail.com

**Keywords:** cell-penetrating peptides, penetratin, aza-amino acid, aza-peptide, flow cytometry

## Abstract

The cell-penetrating peptide (CPP) penetratin has gained much attention over many years due to its potential role as a transporter for a broad range of cargo into cells. The modification of penetratin has been extensively investigated too. Aza-peptides are peptide analogs in which one or more of the amino residues are replaced by a semicarbazide. This substitution results in conformational restrictions and modifications in hydrogen bonding properties, which affect the structure and may lead to enhanced activity and selectivity of the modified peptide. In this work, the Trp residues of penetratin were substituted by aza-glycine or glycine residues to examine the effect of these modifications on the cellular uptake and the internalization mechanism. The substitution of Trp^48^ or Trp^48,56^ dramatically reduced the internalization, showing the importance of Trp^48^ in cellular uptake. Interestingly, while aza-glycine in the position of Trp^56^ increased the cellular uptake, Gly reduced it. The two Trp-modified derivatives showed altered internalization pathways, too. Based on our knowledge, this is the first study about the effect of aza-amino acid substitution on the cell entry of CPPs. Our results suggest that aza-amino acid insertion is a useful modification to change the internalization of a CPP.

## 1. Introduction

The use of therapeutics and imaging agents is frequently impeded by their limited ability to permeate cell membranes, a challenge attributed to the hydrophilic nature and size of biologically active molecules. Such drawbacks require the use of vast amounts of therapeutics or imaging agents to achieve the desired effect. To overcome this obstacle there are some possibilities. For instance, the incorporation of fluorine and unnatural modification in peptide chemistry enhance the permeability [1]. The substitution of an amide by thioamide in a macrocyclic peptide also improves the permeability and bioavailability of these peptides when administered through the oral route in rats [2]. Peptide-based nanoparticles [3,4], in their turn, play a crucial role to overcome this drawback too. Another important finding with Fmoc-FF nanogels revealed their selectivity toward cancer cell lines overexpressing the protein caveolin1, and effectively engaging in caveolae-mediated endocytosis [5].

A particular type of peptides with a maximum of 30 amino acids, known as cell-penetrating peptides, can be used to improve the cellular uptake of cargos [6,7]. CPPs are essential molecular transporters that can cross plasma membranes with low cytotoxicity.

As carriers to deliver biologically active compounds, cell-penetrating peptides have undoubtedly demonstrated their value in transporting several kinds of molecules, e.g., peptides [8,9], proteins [10], small drug molecules [11], and oligonucleotides [12]. The modification of CPPs has been a focus of research over the past several years through peptidomimetics [7], the mimics of natural peptides, where the biological effect is retained while simultaneously improving any unfavorable properties such as poor bioavailability, rapid metabolism, and insufficient receptor selectivity and enhancing the pharmacokinetic and pharmacological properties [13].

**Figure 1:**
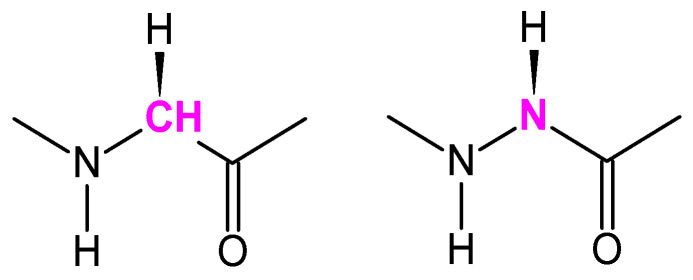
The structure of glycine and aza-glycine moiety.

Among the numerous structural modifications for developing peptide mimics [14,15], the replacement of one or more amino acids by an aza-amino acid in which C_α_ is replaced by nitrogen produces an aza-peptide (Figure 1) [16].

The synthesis of building blocks of peptides containing aza-amino acid residues requires the use of hydrazine. Therefore, the combination of hydrazine and peptide chemistry is important for aza-peptide synthesis. The replacement of a natural amino acid with its counterpart, aza-amino acid residue, imparts special conformational properties compared with the original peptide structure due to the loss of asymmetry and reduction of flexibility.

Aza-peptides have been shown by spectroscopic [17,18], computational [17,19], and crystallographic [20,21] studies to induce turn conformations in the parent peptides. In addition, this exchange may enhance the biological activity and the pharmacokinetic and pharmacological properties of the original peptide.

Furthermore, it may improve stability toward chemical and enzymatic degradation [22,23,24]. One of the most important potent aza-peptide inhibitors of the HIV protease is atazanavir, U.S. Food and Drug Administration (FDA)-approved as an antiretroviral drug [25]. The aza-analogues of the linear decapeptide luliberin were prepared, and the most active was around 100 times more potent than the parent peptide [26]. In particular, one aza-peptide from the series was approved for use as a drug in the treatment of prostate cancer (Zoladex) [27]. Furthermore, the replacement of glycine by aza-glycine in the peptide motif Arg-Gly-Asp (RGD), responsible for cell adhesion, shows that this exchange may affect both activity and selectivity [28].

Penetratin, a 16-amino-acid-long peptide derived from the Antennapedia homeodomain, was among the first CPPs discovered in 1988 [29]. Since that time, its mechanism of entry into cells and the possible modifications of its sequence have been intensively investigated. An earlier study revealed that penetratin and the dodeca-penetratin derivative with 12 amino acids had similar translocational efficacy on HeLa, L929, and RAW cells [30]. This similarity highlights the relevance of basic residues and aromatic tryptophans in penetratin internalization and demonstrates that the binding of these cationic peptides to the cell surface is completely nonspecific. In addition, this study reveals the crucial role of the aromatic Trp residue, where the cellular uptake is reduced when this residue is replaced by Phe in position 6 or 14. Walnart et al. showed that the substitution of Trp by Leu in the arginine-rich peptide RRWWRRWRR diminished its uptake [31]. In contrast, our group revealed that one or two Trp, as an aromatic amino acid alone, did not improve the internalization of tetraarginine, but it was very efficient in the presence of the Dabcyl group (4-((4-(dimethylamino)phenyl)azo)benzoyl group) [32]. It seems that some kind of balance between the hydrophobicity/aromaticity and positive charge is necessary for efficient internalization [33]

Penetratin (R^43^QIKIWFQNRRMKWKK^58^), as a well-known cell-penetrating peptide, has a high capability to drive the internalization of cargos across the cell membrane [34,35,36]. It can do this at both 4 and 37 °C and reach the cytoplasm and nucleus, where it can be recovered without apparent degradation [29]. Certain amino acids in its sequence significantly impact internalization. The hydrophobic amino acid tryptophan (Trp) has a crucial role in its internalization [37,38]. When it was replaced by Phe in position 48, the internalization was reduced [39]. After both Trp residues were substituted by Phe, the modified peptide altered lipid interactions and hindered translocation into live cells [29,40,41,42]. The shortening of the penetratin resulted in an efficient dodecapenetratin derivative containing 12-amino acids [30]. In the same study, the Phe48- and Phe56-penetratin revealed reduced cellular uptake, which is evidence of the beneficial presence of Trp. It was demonstrated that lipid rafts are involved in peptide internalization, and the basic residues of the peptide play a significant role in the internalization. The shortest penetratin derivative is the peptide ^52^Arg-Arg-Met-Lys-Trp-Lys-Lys^58^, which was necessary and sufficient for the internalization [43], and it could deliver peptide cargo efficiently into HeLa cells [36]. Based on this, Trp is considerably advantageous but not indispensable for cellular uptake. Recently, the modification of the peptide backbone with semicarbazide has gained much attention due to the diversity of biological and pharmacokinetic properties of the resulting aza-peptide. In particular, aza-glycine incorporation is widely applied due to its simplicity and high tolerability. For instance, improved metabolic stability and excellent agonistic activity have been reported for the truncated decapeptide ligand-targeted KISS1R receptor in case of the replacement of glycine by analog aza-Gly at position 51 [44].

This paper describes the synthesis and characterization of various penetratin derivatives. In the first set, Trp residue was replaced by one aza-Gly or Gly near the C-termini or near the N-termini to study the influence of this substitution on the internalization [(*Cf*-RQIKIWFQNRRK-azaGly-KK-*NH*_2_, *Cf*-RQIKI-azaGly-FQNRRKWKK-*NH*_2_, *Cf*-RQIKIWFQNRRKGKK-*NH*_2_, *Cf*-RQIKIGFQNRRKWKK-*NH*_2_]. The effect of two aza-Gly or Gly in both positions (6 and 13) was also investigated using peptides *Cf*-RQIKI-azaGly-FQNRRK-azaGlyKK-*NH*_2_ and *Cf*-RQIKIGFQNRRKGKK-*NH*_2_. To measure the cellular uptake, the N-terminus of peptides was labelled o by 5(6)-carboxyfluorescein (Cf) on a solid support. The cellular uptake was examined on the A-431 cell line by flow cytometry. Based on our results, we assume that with our modification, the cellular uptake and the stability of the penetratin peptide can be enhanced.

## 2. Materials and Methods

### 2.1. Synthesis of Peptides

All peptides were synthesized manually by solid-phase peptide synthesis on Rink amide MBHA resin (0.1 g, 0.65 mmol/g) using Fmoc/*t*Bu strategy. To protect the side chain of arginine and lysine 2,2,4,6,7-pentamethyldihydrobenzofuran-5-sulfonyl (Pbf) and *tert*-butyloxycarbonyl (Boc) group were used, respectively. Side chains of glutamine and asparagine were protected by the triphenyl methyl (Trt) group. The Fmoc temporary protecting group for the N-terminal amine group was removed by using a solution containing 2% piperidine and 2% 1, 8-diazabicyclo [5.4.0] undec-7-ene (DBU) in N,N-dimethylformamide (DMF) (2 + 2 + 5 + 10 min) based on the standard protocol, followed by extensive washing (8 × 1 min) with DMF. Three equimolar excesses of both N,N-diisopropylcarbodiimide (DIC) and ethyl(hydroxyimino)cyanoacetate (OxymaPure) coupling reagents were added to three equimolar excesses of each Fmoc-protected amino acids dissolved in DMF for carrying coupling reactions for 60 min at room temperature. The coupling of aza-Gly was carried out using 1,1′-carbonyldiimidazole and Fmoc-Hydrazide dissolved in DMF and in the presence of 2 eq. of N,N-Diisopropylethylamine (DIEA) [45]. The efficiency of coupling was monitored by ninhydrin test. The success of the next amino acid’s coupling to the aza-Gly could not be detected by Kaiser test. Therefore, this amino acid derivative was coupled twice. After removing the last N^α^-Fmoc group from the peptide resin, we attached the 5(6)-carboxyfluorescein (Cf) to the N-terminal amino acid using the same coupling reagents. The resulting peptides were cleaved from the resin using a mixture containing 0.365 g phenol, 5 mL TFA, 0.25 mL distilled water, 0.25 mL thioanisole, and 0.125 mL 1,2-ethanedithiol. The crude products were precipitated using dry diethyl ether, then were dissolved in 10% acetic acid, and freeze-dried. The crude peptides were purified by semi-preparative RP-HPLC, their purity was checked by analytical RP-HPLC, and they were identified by ESI-MS (Supplementary Materials, Figures S1–S14).

### 2.2. Determination of Cellular Uptake by Flow Cytometry

To investigate the cellular uptake of peptides, 10^5^ cultured A-431 cells (human skin squamous cancer cells, which was a generous gift from Prof. József Tóvári (Institute of Oncology, Budapest, Hungary)) per well were plated on 24-well plates. The cells were incubated for 24 h at 37 °C. The cells were treated with a serum-free medium as a negative control and with peptide solutions in 5 µM concentration in a serum-free medium for 90 min. After the incubation, the cells were washed, and 100 µL of trypsin was applied for 10 min to eliminate peptides adhered to the membrane and to detach the cells from the plates. Trypsin activity was stopped by adding 800 µL of HPMI buffer (glucose, NaHCO_3_, NaCl, HEPES, KCl, MgCl_2_, CaCl_2_, Na_2_HPO_4_·2H_2_O) containing 10% fetal bovine serum. Cells were then transferred from the plates to FACS tubes. Centrifugation of the cells took place at 216× *g* at 4 °C for 5 min, and the supernatant was removed. Resuspension of the cells was performed by 250 µL HPMI, followed by fluorescence intensity measurements using a flow cytometer (BDLSR II, BD Bioscience, San Jose, CA, USA). The analysis of the data was performed using FACSDiVa 5.0 software. To study the effect of inhibitors, cells were pretreated with the solution of inhibitors for 30 min, followed by the treatment with the peptide solution at 5 µM for 90 min. Inhibition of macropinocytosis was performed by using 5-(N-Ethyl-N-isopropyl)amiloride (EIPA) (500 µM) [46], of clathrin-mediated endocytosis by using chlorpromazine (CPZ) (300 µM) [47]. The caveolae/lipid raft-mediated endocytosis was prevented with methyl-β-cyclodextrin (mBCD) (36 mM) [48], and colchicine (Col) (200 mM) was employed to determine the role of microtubules and consequently, the significance of pinocytosis [49]. To inhibit all endocytosis routes, sodium azide (NaN_3_) (500 µM) and 2-Deoxyglucose (DOG) (250 mM) was used. 

### 2.3. Calculations

To investigate the structural properties of the model peptides, Pen(desMet): RQIKIWFQNRRKWKK and Trp56GlyPen(desMet): RQIKIWFQNRRK**G**KK, we employed Pep-Fold4 that can predict peptide structures from amino acid sequences [50] to generate the initial structures of model peptides at pH 7.5 and 100 mM ionic strength. We chose the top best structure among the five best structures of model peptide (Supporting Materials Figure S17). For Trp56aGlyPen(desMet) (RQIKIWFQNRRK-azaGly-KK), we modified the top best structure of Trp56GlyPen(desMet) by replacing alpha carbon (C_α_H) with a nitrogen atom. These initial structures of model peptides were fully optimized at the ωB97XD/3-21G* level of theory using the program Gaussian 16 [51]. The ωB97XD functional is a range-separated version of Becke’s 97 functional with additional dispersion correction [52]. Due to the large system of model peptides, we used the smaller basis set 3-21G*. The secondary structure of the optimized structures was analyzed using the DSSP website (https://swift.cmbi.umcn.nl/gv/dssp/, accessed on 25 January 2024) [53].

### 2.4. Statistical Analysis

The finding results of the cellular uptake studies are represented by the mean value ± standard deviation. For the statistical analysis, the Student’s *t* test was applied. *p* values less than 0.05 were considered statistically significant results.

## 3. Results

### 3.1. Design and Synthesis of Peptides

Based on these findings, we planned to synthesize a set of penetratin derivatives to investigate the effect of the Trp substitution by aza-glycine and glycine at position 48 or 56 or both on the cellular uptake. These peptides were synthesized manually on a Rink-amide MBHA resin using solid-phase peptide synthesis by Fmoc/*t*Bu strategy. All the natural amino acids were coupled using DIC and Oxyma pure as coupling reagents. The N-terminus of the peptides were labelled by 5(6)-carboxyfluorescein and the same reagents on the solid phase. The aza-Gly residue was inserted into the sequence using three equivalents of Fmoc-NH-NH_2_ and three equivalents of 1,1-carbonyldiimidazole (CDI) in the presence of DIEA as a base [45]. The success of the coupling reactions was examined by the ninhydrin test. The coupling of the amino acid to the aza-Gly cannot be detected by the ninhydrin test, therefore, we performed its coupling twice. The peptides were cleaved from the resin using a cleavage mixture. The crude peptides were purified by RP-HPLC and characterized by analytical RP-HPLC and ESI-MS (Table 1; the analytical RP-HPLC chromatograms and MS spectra are in the Supplementary Materials, Figures S1–S14.

### 3.2. Cellular Uptake

The cellular uptake of the labelled penetratin derivatives (Table 1) and of penetratin was measured by flow cytometry on A-431 human skin squamous cancer cells.

The cells were treated with a solution of peptides at 5 µM concentration at 37 °C for 90 min. The measured fluorescence values were corrected with the autofluorescence of the untreated cells. To determine the toxicity of the conjugates, the live/dead cell ratio was calculated. According to this, none of the conjugates exhibited cytotoxicity at this concentration (Supplementary Materials Figure S15)

**Figure 2:**
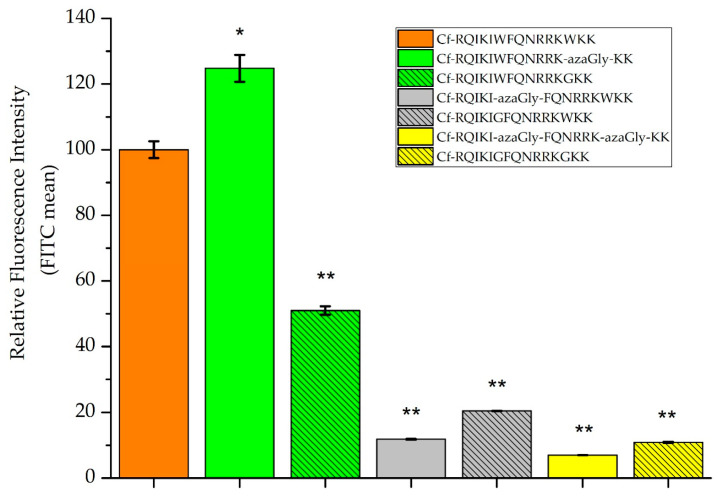
Comparison of the cellular uptake of the labelled penetratin derivatives into A-431 cells. Cells were incubated with 5 µM peptide solution for 90 min at 37 °C. The fluorescence intensity of the cells was determined by flow cytometry. The fluorescence intensity is relative to Pen(desMet) at 5 µM (100%). Data represent the mean ± standard deviation (SD). Any significant difference to the control was measured using the Student’s *t* test. The asterisks show a significant difference between the control Pen(desMet) and its derivatives (* *p* < 0.05, ** *p* < 0.01).

The results revealed that peptide Trp56aGlyPen(desMet) had higher internalization (124.8%) than the Pen(desMet) (100%), and it is 2.4-fold higher than those of the peptide Trp56GlyPen(desMet) (50.0%). However, the substitution of Trp residue near the N-terminus (Trp48aGlyPen(desMet) and Trp48GlyPen(desMet)) or in both positions (Trp48,56aGlyPen(desMet) and Trp48,56GlyPen(desMet)) decreased markedly the internalization, which varied between 6% and 20.5% (Figure 2).

### 3.3. Study of the Different Endocytic Pathways of Internalization

**Figure 3:**
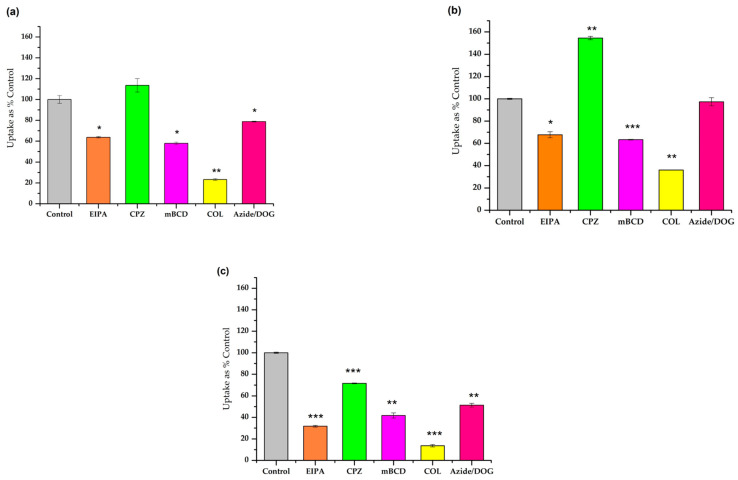
Effect of endocytosis inhibitors on the internalization of peptides (**a**) Pen(desMet), (**b**) Trp56aGlyPen(des), and (**c**) Trp56GlyPen(des). A-431 cells were pretreated with the EIPA (500 µM), CPZ (300 µM), mBCD (36 mM), COL (200 mM), NaN_3_ (500 µM), and DOG (250 mM) for 30 min, followed by treatment with peptides (5 µM) for 90 min. Any significant difference from the control was determined by Student’s *t*-test (* *p* < 0.05). Data represent the mean ± standard deviation (SD).The asterisks show a significant difference between the control Pen(desMet) and its derivatives (* *p* < 0.05, ** *p* < 0.01, *** *p* < 0.001).

We further investigated the cellular uptake mechanism of the most promising peptides (Pen(desMet), Trp56aGlyPen(desMet), and Trp56GlyPen(desMet)) using different inhibitors on the A-431 cell line (Figure 3). The following endocytic inhibitors were used: EIPA (5-(N-ethyl-N-isopropyl) amiloride) as an inhibitor of macropinocytosis, chlorpromazine (CPZ) as an inhibitor of a clathrin-mediated endocytosis, and the cholesterol-depleting reagent methyl-beta-cyclodextrin (mBCD) was used to inhibit the caveolae-mediated endocytosis. Finally, colchicine (COL) was applied to inhibit microtubule formation.

In addition, the A-431 cells were pretreated with sodium azide (NaN_3_) and 2-deoxyglucose (DOG) to study the energy-independent pathways. This pretreatment results in ATP depletion and therefore the inhibition of energy-dependent pathways (Figure 3).

The effect of EIPA, mBCD, and COL on the cellular uptake was the same on all peptides. They inhibited the uptake significantly (14–68%). It is worth mentioning that the Gly-substituted derivative was more sensitive towards these inhibitors (32%, 42%, and 14%, respectively) (Figure 3c). The CPZ had a very divergent effect. While it did not have any influence on the internalization of Pen(desMet), it decreased the cellular uptake in the case of Trp56GlyPen(des) and had an increasing effect on the internalization of Trp56aGlyPen(des) derivative.

Upon the pretreatment with NaN_3_ and DOG, the results revealed a significant decrease in the uptake of Trp56GlyPen(des) (50% of the untreated control), moderate influence in the case of Pen(desMet) (79%), and no effect on the uptake of Trp56aGlyPen(des) (97%).

### 3.4. Calculation

**Figure 4:**
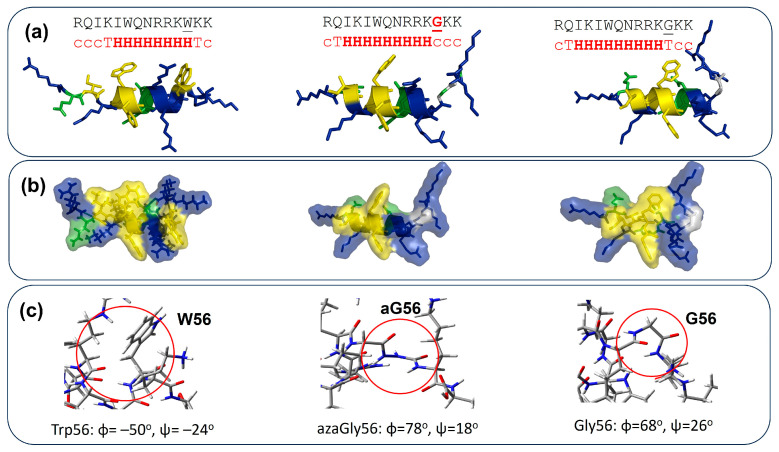
Structural properties of the most promising peptides, Pen(desMet) (left), Trp56aGlyPen(des) (middle), and Trp56GlyPen(des) (right). (**a**) Primary structure of peptides. The unlined G (with red color) represents azaGly residue. The 2-dimensional secondary structure of ωB97XD-optimized structures for the model peptides is analyzed through the DSSP website (c represents coil, T turns, and H is helical structure). (**b**) Surface model of the predicted. The color code for the amino acid residue: hydrophobic residue—yellow; polar residue—green; hydrophilic residue—blue. (**c**) The dihedral angle of amino acid residue at position 56 of the model peptides. Red circle represents the amino acid residue, W56, azaGly56, or Gly56.The coordinates of three optimized peptide structures are found in the Supporting Materials Table S1.

We investigated the structural properties of the most promising peptides (Pen(desMet), Trp56aGlyPen(des), and Trp56GlyPen(des)) at the wB97XD functional with a 3-21G* basis set. The optimized structure of these peptides adopts the helical structure with different lengths and positions (Figure 4a). Interestingly, Trp56aGlyPen(des) and Trp56GlyPen(des) adopt a longer helical structure than the Pen(desMet) peptide. The surface of the model peptides was examined, showing that the modification of amino acid at position 56 would change the hydrophobic (middle) and hydrophilic (C-terminal) surface area of the peptides (Figure 4b). Also, the backbone dihedral angles of amino acid residue at the 56 position are located in the right-handed helical region (W56: ϕ = −50^o^, y = −24^o^), left-handed helical region (G56: ϕ = +68^o^, ψ = +26^o^), and the bridge region (azaGly56: ϕ = +78^o^, ψ = +18^o^). The results show that incorporating azaGly residue might change the C-terminal region of Pen(desMet) peptide because azaGly residue adopts the limited conformational space [54].

## 4. Discussion

The amphipathic, positively charged penetratin is one of the most widely studied CPPs. This is due to the exceptional capability of this peptide across biological membranes. It is supposed that the binding affinity of these CPP is due to two main interactions: the long-range coulombic attraction occurs between the positively charged basic residues (arginines and lysines) and the negatively charged parts of the membrane [55,56,57], while the non-specific electrostatic attraction is caused by the cation–π interactions between the positively charged components of membrane lipids and the π face of aromatic side chains of the hydrophobic amino acid tryptophan and phenylalanine [58]. Christiaens et al. studied the contribution of the basic residues to the cellular uptake through the double substitutions of Lys and/or Arg residues to Ala within penetratin (residue numbers 43–58) [38]. Their finding showed the essential role of the positively charged residues in the peptide’s initial electrostatic interaction with negatively charged phospholipid vesicles. The role of the Trp residues in penetratin and its variants has been intensively investigated. Derossi et al. [29] demonstrated that the substitution of Trp by Phe (W48F/W56F-penetratin) reduced the peptide internalization, highlighting the importance of Trp residues in translocation. This finding was confirmed later by Letoha et al. [30].

Aza-peptides are attractive targets as bioactive agents, because, in their analogs, the aza-substitution has resulted in better activity and selectivity, as well as other enhanced properties such as prolonged duration of action and metabolic stability [59,60].

Based on these, we designed penetratin derivatives with aza-glycine. Penetratin was selected because of its well-known structure-internalization relationships. Earlier we proved that Met can be removed without losing the cell-penetrating ability [61], and thus, the potential problem with Met can be avoided. To obtain new insights into the effects of the structure on the internalization, in our derivatives, the Trp residues were substituted with aza-glycine. Many studies proved the positive effect of Trp residues in the internalization of penetratin [58]. It is supposed that its aromatic ring is crucial in the interaction with the cell-membrane. We wondered whether this interaction is the only effect or whether it also affects the structure of the peptide. Thus, six derivatives with one or two glycine or aza-glycine were synthesized. Both Trp residues were substituted one by one and together. Both substitutions resulted in the loss of side chain, while the presence of aza-glycine may induce altered peptide conformation. This possible change can provide not only stability and rigidity to the peptide backbone but may also enhance the cellular uptake. In addition, both replacements decrease the hydrophobicity of the penetratin variants.

The replacement of Trp48 (Trp48aGlyPen(desMet) and Trp48GlyPen(desMet)) dramatically decreased the cellular uptake on A-431 cells (Figure 2). As both substitutions (Gly and azaGly) had the same effect, we can say that the possible structural changes caused by the aza-peptide backbone could not balance the loss of the aromatic side chain. In contrast to this, the effect of the Trp56 substitution was strongly dependent on the substituent. The presence of the aza-Gly residue (Trp56aGlyPen(des)) increased the cellular uptake by 20%, while the Gly substituent (Trp56GlyPen(des)) made a 50% decrease in the internalization (Figure 2). In the literature, there are contradictory results about the role of the two Trp residues in the internalization. In comparison with other homeodomain-derived CPPs, penetratin is the only one that has Trp56. While the other derivatives contain only the Trp48, they have the same or better internalization [62]. The deletion of Trp48 and Phe49 also resulted in abolished internalization [63]. Unfortunately, other results obtained by different substitutions are very contradictory. The mutation of Trp at positions 48 and 56 by Ala and Phe was studied [29,43,64,65]. Using Gly or aza-Gly as a substituent of Trp results in the elimination of the side chain without changing the peptide backbone size. The main difference between them is that aza-Gly may induce a secondary structure. Our results suggest that the Trp side chain may have a role in inducing or stabilizing the special conformation, which facilitates the internalization. That can be why the aza-Gly substitution not only retained but increased the efficiency of the cellular uptake. Its presence may induce a secondary structure, affecting the membrane association and the internalization mechanism. This conformation is due to the substitution of an amino acid residue for a semicarbazide in a peptide which reinforces the formation of β-turn conformation. To investigate this possible role of aza-Gly moiety, the solution phase structure of Trp56aGlyPen(des) and Trp56GlyPen(des) was studied by ECD. According to the minima at and below 200 nm, the two mutant peptides had the same unordered structure in the PBS buffer than the penetratin had. In the presence of 50% TFE, negative peaks appear at 206 and 224 nm. This suggests that under these conditions an alpha helical conformation also occurs but all spectra have the same form [66] (Supplementary Materials Figure S16).

We also examined the possible structural changes caused by the substitutions using density functional theory (DFT). DFT calculation predicted that the Pen(desMet) peptide adopted a helical structure from residue I49 to residue W56. Since it has been reported that the Gly residue is flexible compared with the Trp residue, structural changes in the Trp56Gly peptide is expected. Interestingly, Trp56Gly substitution enhanced the helical structure from I^45^KIWFQNRRK^54^ but not Gly56. Trp56aGly substitution is similar to the Trp56Gly overall, but there are some conformational changes at the C-terminal region (Figure 4b).

While the replacement of Trp48 caused the elimination of cellular uptake independent of the replacing amino acid residue, the aza-Gly substitution of Trp56 increased the internalization. Thus, the effect of two substitutions was also studied. The disubstituted derivatives (Trp48,56aGlyPen(desMet) and Trp48,56GlyPen(desMet)) showed similar weak internalization as the Trp48aGlyPen(desMet) and Trp48GlyPen(desMet). Based on these results, it can be stated that the substitution of the N-terminal Trp residue (Trp^48^) decreased the internalization and that the Trp^48^ has a higher influence on the internalization than the Trp^56^. Its elimination overwrites the positive effect of aza-Gly at position 56. This is in good agreement with those earlier results, which showed the more important role of Trp^48^ in internalization [29,63]. Although, later it was found that the double mutant derivative (W48FW56F) has slightly lower and more strongly cell line-dependent internalization than the penetratin [30]. Comparing these results to our findings, the penetratin derivative with two Phe residues is more efficient than our derivatives with two glycines or two aza-glycines. As the phenylalanine is a hydrophobic amino acid, it seems that the presence of hydrophobic amino acid residues is crucial for better internalization.

The pathway of the internalization of penetratin and its derivatives was extensively examined. Many studies have shown that endocytosis is the major cellular uptake mechanism and different routes were described. While some reported micropinocytosis on CHO-K1 cells [67], another study did not show any dependence of penetratin internalization on EIPA treatment in the case of HeLa cells, which refers to the fact that macropinocytosis does not play a role in its cellular uptake [68]. In a recent study, different endocytosis inhibitors were tested and the results revealed that the primary pathway of penetratin internalization might be the caveolae and clathrin-mediated endocytosis, and is typically dependent on ATP [69]. In our study, EIPA inhibited the internalization of Pen(desMet) on A-431 cells, which was 64% of control cells. The methyl-β-cyclodextrin as an inhibitor of caveolae/lipid raft-mediated endocytosis decreased the Pen(desMet) cellular uptake to 58% of the untreated cells. A similar effect of mBCD was published on HeLa, L929, and RAW cells [30]. The influence of COL was the strongest, reducing the cellular uptake to 23%, indicating the important role of the microtubular system in the different kinds of endocytosis. The only inhibitor that did not alter the internalization was CPZ, a clathrin-mediated endocytosis inhibitor. Although these results are contradictory with recently published data [69], the different cell lines and the 10 times higher concentration (50 µM and 5 µM) could explain the difference [70]. Finally, the Azide/DOG treatment caused energy depletion and reduced the cellular uptake of Pen(desMet) (79%). Although this effect is lower than those of other inhibitors, it also shows the ATP dependence of the cellular uptake.

When Trp56 was substituted by Gly (Trp56GlyPen(des)), the effect of the inhibitors was similar to that of Pen(desMet), but their inhibition was higher. The only significant difference was that in the case of Trp56GlyPen(des), the CPZ also had an inhibitory effect. These findings may suggest that the elimination of the side chain of Trp^56^ decreases the possibility of non-endocytic cellular uptake. When aza-Gly was used to substitute the Trp^56^ (Trp56aGlyPen(des)), the EIPA, mBCD, and COL had the same effect as in the case of Pen(desMet) or Trp56GlyPen(des), but CPZ significantly increased the cellular uptake, while azide/DOG treatment did not have any effect on it. As the effects of Gly and azaGly are very different on both the cellular uptake and the internalization pathway, we can say that the structural difference of derivatives induced by the aza-amino substitution can be the reason for it.

Based on our knowledge, this is the first study that examined the effect of aza-amino acid substitution on the cellular uptake of cell-penetrating peptides. This useful modification was used earlier in the case of peptides whose biological activity strongly depend on their structure, e.g., receptor ligands or inhibitors. Unfortunately, in the case of CPPs, we have little knowledge about what is or even if there is any structural requirement for the cellular uptake. Our results suggest that aza-glycine can be used to modify a peptide to retain its biological activity. Although we lose the side chain, the modified peptide backbone can balance it.

## Figures and Tables

**Table 1:** Chemical Characterization of the Peptides.

Sequence	Code	R_t_ ^a^	M_calc_	M_meas_ ^b^
Cf-RQIKIWFQNRRKWKK	Pen(desMet) ^c^	15.1	2472.9	2472.2
Cf-RQIKIWFQNRRK-azaGly-KK	Trp56aGlyPen(desMet)	14.8	2344.7	2344.2
Cf-RQIKI-azaGly-FQNRRKWKK	Trp48aGlyPen(desMet)	13.4	2344.7	2344.1
Cf-RQIKI-azaGly-FQNRRK-azaGly-KK	Trp48,56aGlyPen(desMet)	12.0	2216.5	2216.2
Cf-RQIKIWFQNRRKGKK	Trp56GlyPen(desMet)	15.0	2343.7	2343.2
Cf-RQIKIGFQNRRKWKK	Trp48GlyPen(desMet)	13.6	2343.7	2343.2
Cf-RQIKIGFQNRRKGKK	Trp48,56GlyPen(desMet)	13.2	2214.5	2214.1

^a^ The analytical chromatogram was obtained using Hypersil Hypurity C18 column (4.6 mm × 150 mm, 5 µm, 190 Å). Linear gradient elution (0 min 0% B, 2 min 0% B, 22 min 90% B) was used at 1 mL/min flow rate. The absorbance was measured at λ = 220 nm. ^b^ The mass spectrometric analysis was conducted on a Bruker Amazon SL (Bremen, Germany). The samples were dissolved in acetonitrile-water (50:50, *v*/*v*), containing 0.1% formic acid. ^c^ Pen(desMet) refers to a penetratin derivative which does not have Met in its sequence.

## Data Availability

Data are contained within the article and Supplementary Materials.

## Supplementary Materials

The following supporting information can be downloaded at: https://www.mdpi.com/article/10.3390/pharmaceutics16040477/s1, Figures S1–S7: HPLC chromatogram of compounds; Figures S8–S15: MS Spectrum of compounds; Figures S15: Cytotoxicity of the peptides; Figures S16: ECD spectra of peptides; Figures S17: PEP-Fold4-generated initial structure of peptides. Table S1: Coordinates of optimized structures for model peptides.
