# Supplementary material for: Influence of Aza-Glycine Substitution on the Internalization of Penetratin
Source: Pharmaceutics. 2024 Mar 30;16(4):477. doi: 10.3390/pharmaceutics16040477 (PMC11053488; doi:10.3390/pharmaceutics16040477)
Supplement: Supplementary file 1 [file pharmaceutics-16-00477-s001.zip › pharmaceutics-2908460-supplementary.pdf]

Supplementary Materials

Influence of aza-glycine substitution on the internalization of penetratin

Karima Tarchoun[1,2], Dóra Soltész[1,2], Viktor Farkas[3], Ho-Jin Lee[4,5], Ildikó Szabó[6], Zoltán Bánóczi[1,*]

[1]ELTE Eötvös Loránd University, Budapest, Hungary; Institute of Chemistry, Faculty of Science, Pázmány Péter sétány. 1/A, Budapest H-1117, Hungary
[2]Hevesy György PhD School of Chemistry, Institute of Chemistry, ELTE Eötvös Loránd University, Pázmány Péter sétány 1/A, H-1117 Budapest, Hungary
[3]HUN-REN-ELTE Protein Modeling Research Group, Institute of Chemistry, Eötvös Loránd University, Pázmány Péter sétány. 1/A, Budapest, Hungary
[4] Department of Natural Sciences, Southwest Tennessee Community College, Memphis, TN 38015, USA
[5] Division of Natural and Mathematics Sciences, LeMoyne-Own College, Memphis, TN 38126, USA
[6]HUN-REN-ELTE Research Group of Peptide Chemistry, 1117 Budapest, Hungary
* Correspondence: zoltan.banoczi@ttk.elte.hu

1. Materials

All amino acid derivatives, Fmoc protected amino acids, N, N′- diisopropylcarbodiimide (DIC), and Rink-amide MBHA resin were purchased from IRIS BiotechGmbH (Marktredwitz, Germany). 1.8- diazabicyclo[5.4.0]undec-7-ene (DBU), N,N-diisopropylethylamine (DIEA), ethanditiol (EDT), 1, 1′-carbonyldiimidazole (CDI) and thioanisole were obtained from FLUKA (Buchs, Switzerland), while , 5-(N-Ethyl-N-isopropyl) amiloride (EIPA), Colchicine (COL), Sodium azide (NaN3), Deoxy-D-glucose (DOG), phenol, trifluoroacetic acid(TFA), Ethyl cyano(hydroxyimino)acetate (Oxyma Pure), 5(6)-carboxyfluorescein (Cf), and all other chemicals used for biological studies, were supplied from Sigma Aldrich (Budapest, Hungary) products. Methyl-beta-cyclodextrin (CyD), Fmoc-hydrazide and chlorpromazine (CPZ) were acquired from TCI chemicals. Solvents used for the synthesis and purification were obtained from Molar Chemicals Ltd (Budapest, Hungary).

1.1 RP-HPLC

All samples were dissolved in a small amount of eluant B (0.1% TFA in acetonitrile-water (80:20, v/v)) and injected into the analytical RP-HPLC. This was performed on Exformma (Exformma Technology (ASIA) Co., Ltd, Hong Kong, China) HPLC system. The column used was Hypersil Hypurity C18 column (4.6 mm × 150 mm, 5 µm, 190 Å),and linear gradient elution (0 min 0% B; 2 min 0% B; 22 min 90% B) was applied using eluent A (0.1% TFA in water), and eluant B at flow rate of 1mL/min. The wavelength for the peaks detection is λ= 220 nm, for both analytical and preparative RP-HPLC. The crude samples were dissolved in eluant A and a semi-preparative Phenomenex Jupiter C18 column (250 × 10 mm I.D.) with 10 mm silica (300 Å pore size) (Torrance, CA, USA) was used for the purification. The flow rate was 4 mL/min, and linear gradient elution is applied.

1.2 Mass spectrometry

The determination of the molecular weight of peptides-conjugates, was performed by ESI-MS, using Bruker Amazon SL (Germany). The samples were prepared and dissolved in water-acetonitrile solution (50:50) containing 0.1% of formic acid. The injection of the samples was done directly through a syringe pump. Parameters: capillary voltage: 4 kV, nebulizer gas: 10 psi, dry gas: 4 L/min, heated capillary temperature: 250 ºC.

1.3 Cell Culture

A-431 human skin squamous cancer cells (CRL-1555TM) were used for the in vitro analysis. The cells were cultured in Dulbecco's Modified Eagle Medium (DMEM) Containing 10% heat inactivated foetal calf serum (FCS), nonessential amino acids (NEAA), sodium pyruvate (1mM), L-Glutamine (2 mM), 1% nonessential amino acids and 1% penicillin-streptomycin (from 10,000 units penicillin and 10 mg/ml streptomycin).The cells were maintained in plastic tissue culture dishes under conditions of 37 °C temperature and a humidified atmosphere comprising 5% CO2 and 95% air.

2. Chemical Characterization of Peptides

2.1. RP-HPLC

All samples were dissolved in a small amount of eluant B (0.1% TFA in acetonitrile-water (80:20, v/v)) and injected into the analytical RP-HPLC. This was performed on Exformma (Exformma Technology (ASIA) Co., Ltd, Hong Kong, China) HPLC system. The column used was Hypersil Hypurity C18 column (4.6 mm × 150 mm, 5 µm, 190 Å), and linear gradient elution (0 min 0% B; 2 min 0% B; 22 min 90% B) was applied using eluent A (0.1% TFA in water), and eluant B at flow rate of 1mL/min. The wavelength for the peaks detection is λ= 220 nm. In many chromatograms two main peaks are visible. These two peaks come from the two Cf isomers (5-carboxyfluorescein and 6-carboxyfluorescein), because we used the mixture of them as 5(6)-carboxyfluorescein for the synthesis of the fluorescently labelled peptides.

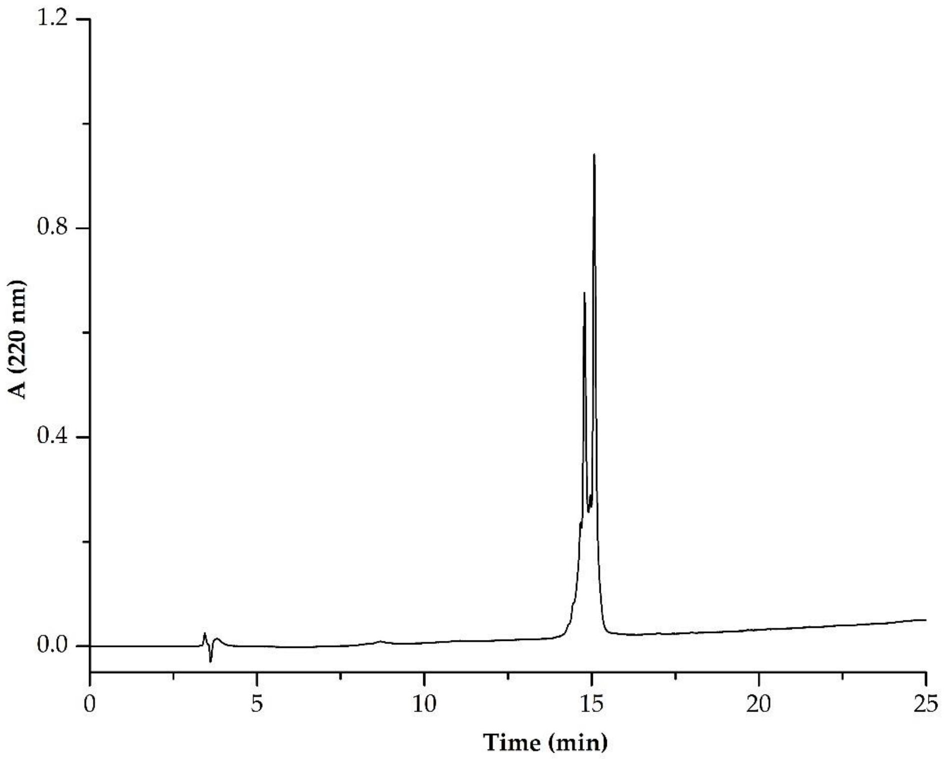

Figure S1. HPLC chromatogram of Cf-RQIKIWFQNRRKWKK-*NH*2. Retention time was obtained on Hypersil Hypurity C18 column (4.6 mm x 150 mm, 5 µm, 190 Å). The applied linear gradient elution was 0 min 0% B, 2 min 0% B, 22 min 90% B at 1 mL/min flow rate. The detection was carried on at λ = 220 nm.

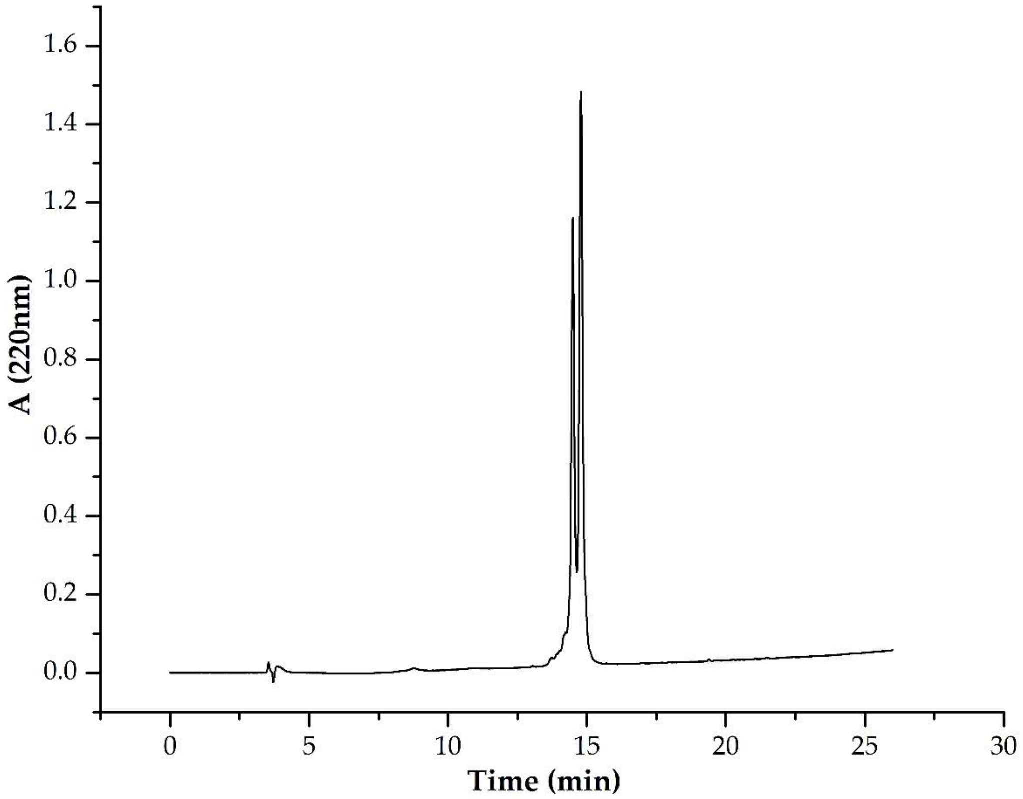

Figure S2. HPLC chromatogram of Cf-RQIKIWFQNRRK-azaGly-KK-*NH*2. Retention time was obtained on Hypersil Hypurity C18 column (4.6 mm x 150 mm, 5 µm, 190 Å). The applied linear

gradient elution was 0 min 0% B, 2 min 0% B, 22 min 90% B at 1 mL/min flow rate. The detection was carried on at λ = 220 nm.

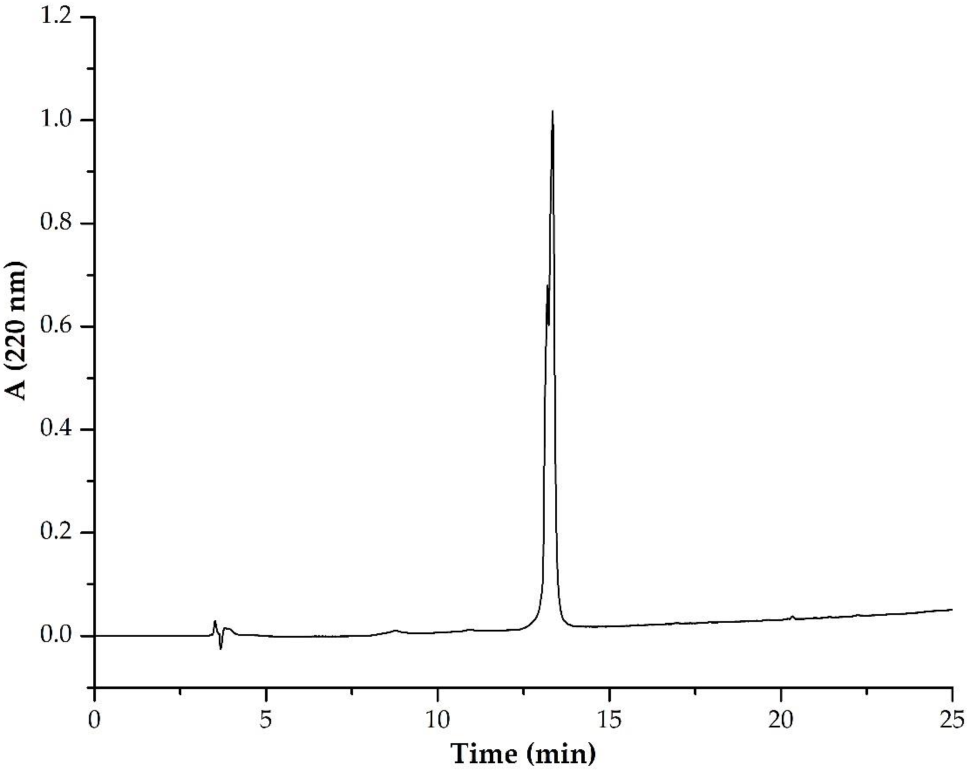

Figure S3. HPLC chromatogram of Cf-RQIKI-azaGly-FQNRRKWKK-*NH*$_2$. Retention time was obtained on Hypersil Hypurity C18 column (4.6 mm x 150 mm, 5 µm, 190 Å). The applied linear gradient elution was 0 min 0% B, 2 min 0% B, 22 min 90% B at 1 mL/min flow rate. The detection was carried on at λ = 220 nm.

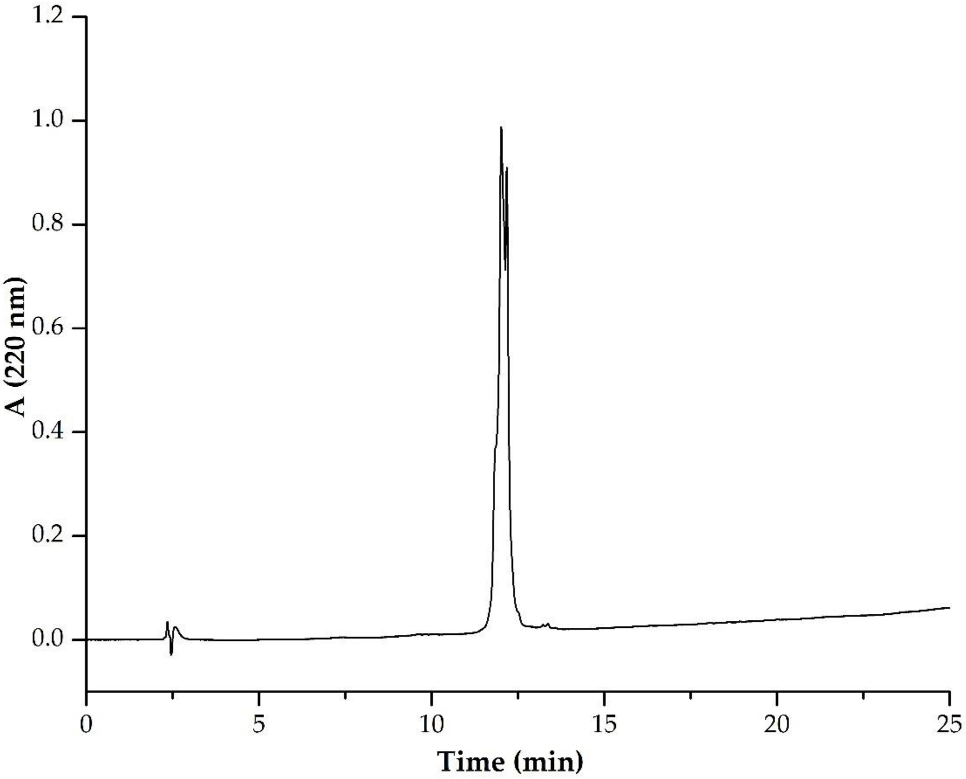

Figure S4. HPLC chromatogram of Cf-RQIKI-azaGly-FQNRRK-azaGly-KK-*NH*2. Retention time was obtained on Hypersil Hypurity C18 column (4.6 mm x 150 mm, 5 μm, 190 Å). The applied linear gradient elution was 0 min 0% B, 2 min 0% B, 22 min 90% B at 1 mL/min flow rate. The detection was carried on at λ = 220 nm.

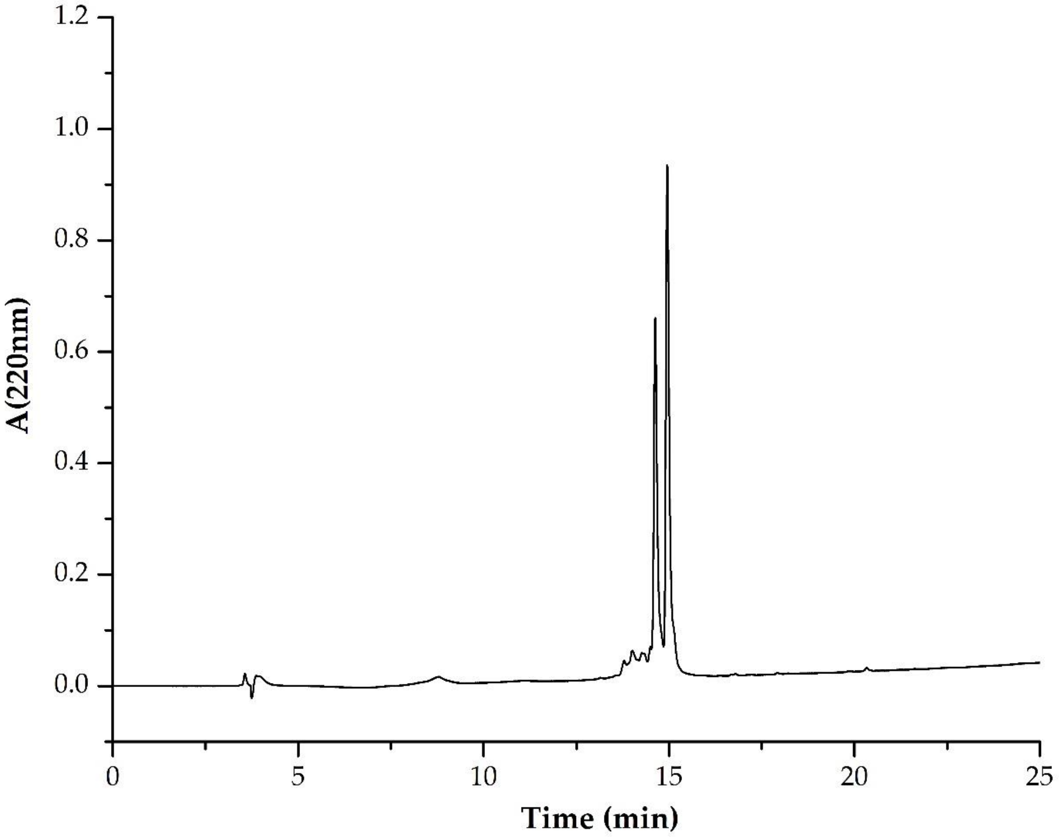

Figure S5. HPLC chromatogram of Cf-RQIKIWFQNRRKGKK-*NH*2. Retention time was obtained on Hypersil Hypurity C18 column (4.6 mm x 150 mm, 5 μm, 190 Å). The applied linear gradient elution was 0 min 0% B, 2 min 0% B, 22 min 90% B at 1 mL/min flow rate. The detection was carried on at λ = 220 nm.

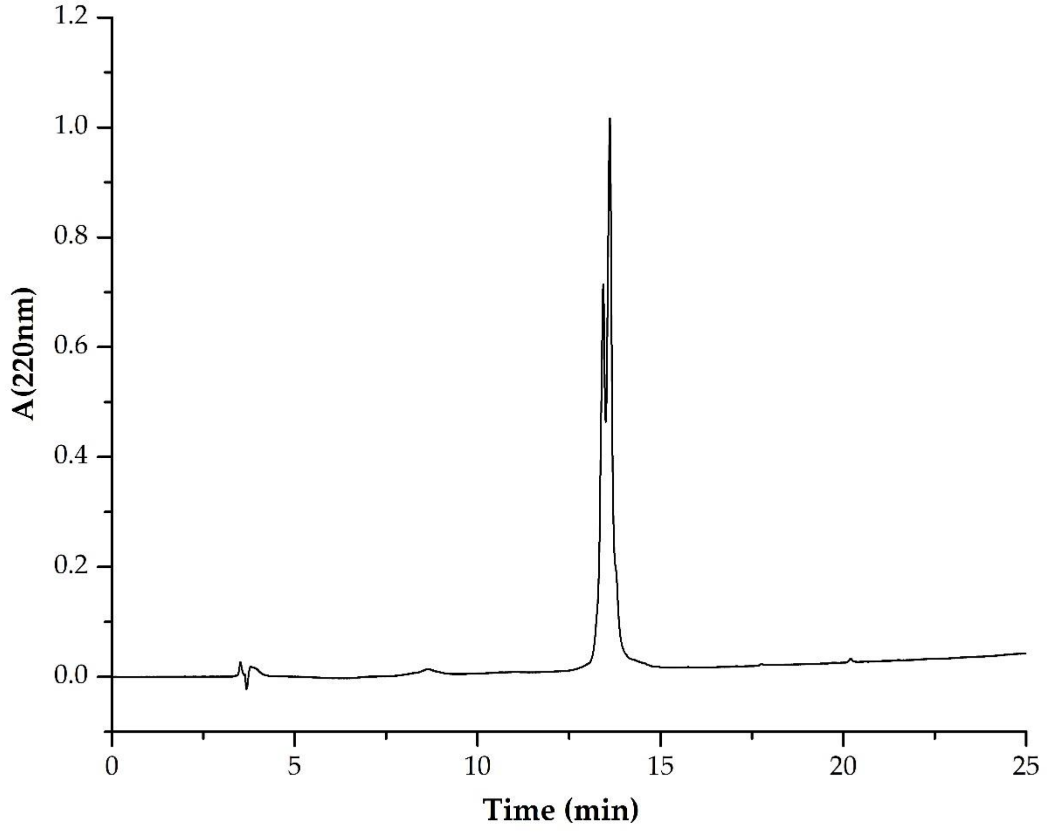

Figure S6. HPLC chromatogram of Cf-RQIKIGFQNRRKWKKNH_2. Retention time was obtained on Hypersil Hypurity C18 column (4.6 mm x 150 mm, 5 μm, 190 Å). The applied linear gradient elution was 0 min 0% B, 2 min 0% B, 22 min 90% B at 1 mL/min flow rate. The detection was carried on at λ = 220 nm.

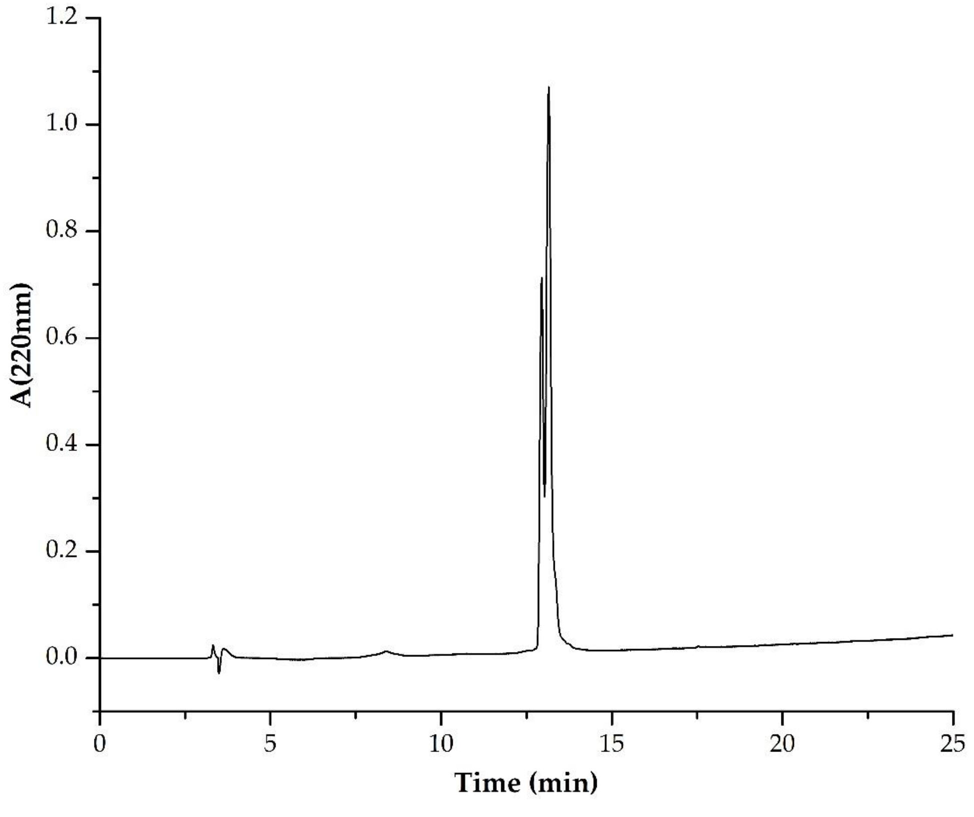

Figure S7. HPLC chromatogram of Cf-RQIKIGFQNRRKGKK-*NH2*. Retention time was obtained on Hypersil Hypurity C18 column (4.6 mm x 150 mm, 5 µm, 190 Å). The applied linear gradient elution was 0 min 0% B, 2 min 0% B, 22 min 90% B at 1 mL/min flow rate. The detection was carried on at λ = 220 nm.

2.2 Mass spectrometry

The determination of the molecular weight of peptides was performed by ESI-MS, using Bruker Amazon SL (Germany). The samples were prepared and dissolved in water-acetonitrile solution (50:50) containing 0.1% formic acid. The injection of the samples was done directly through a syringe pump. Parameters: capillary voltage: 4 kV, nebulizer gas: 10 psi, dry gas: 4 L/min, heated capillary temperature: 250 ºC.

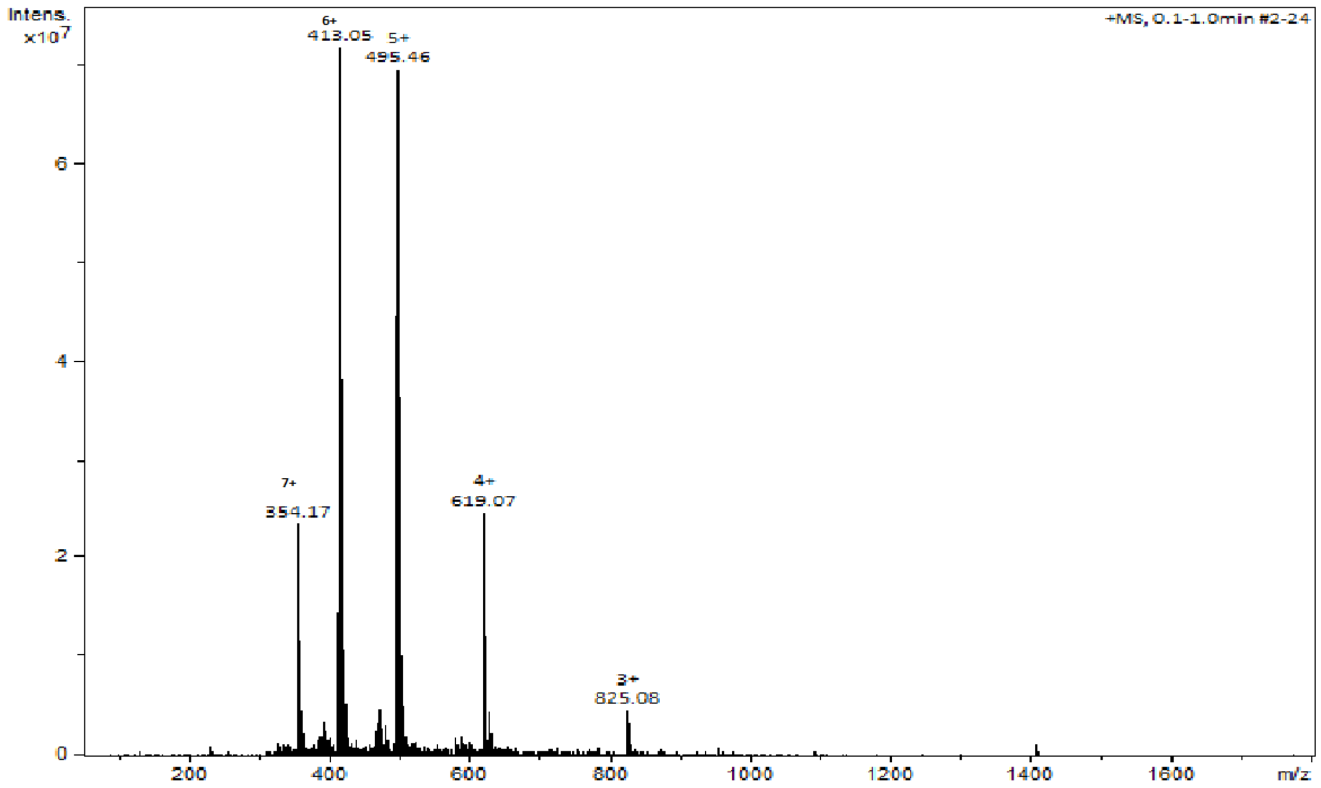

Figure S8. MS Spectrum of Cf-RQIKIWFQNRRKWKK-*NH2*. The identification of the conjugate was determined using Bruker Amazon SL (Germany). The sample is dissolved in water-acetonitrile (50:50) with 0.1% formic acid.

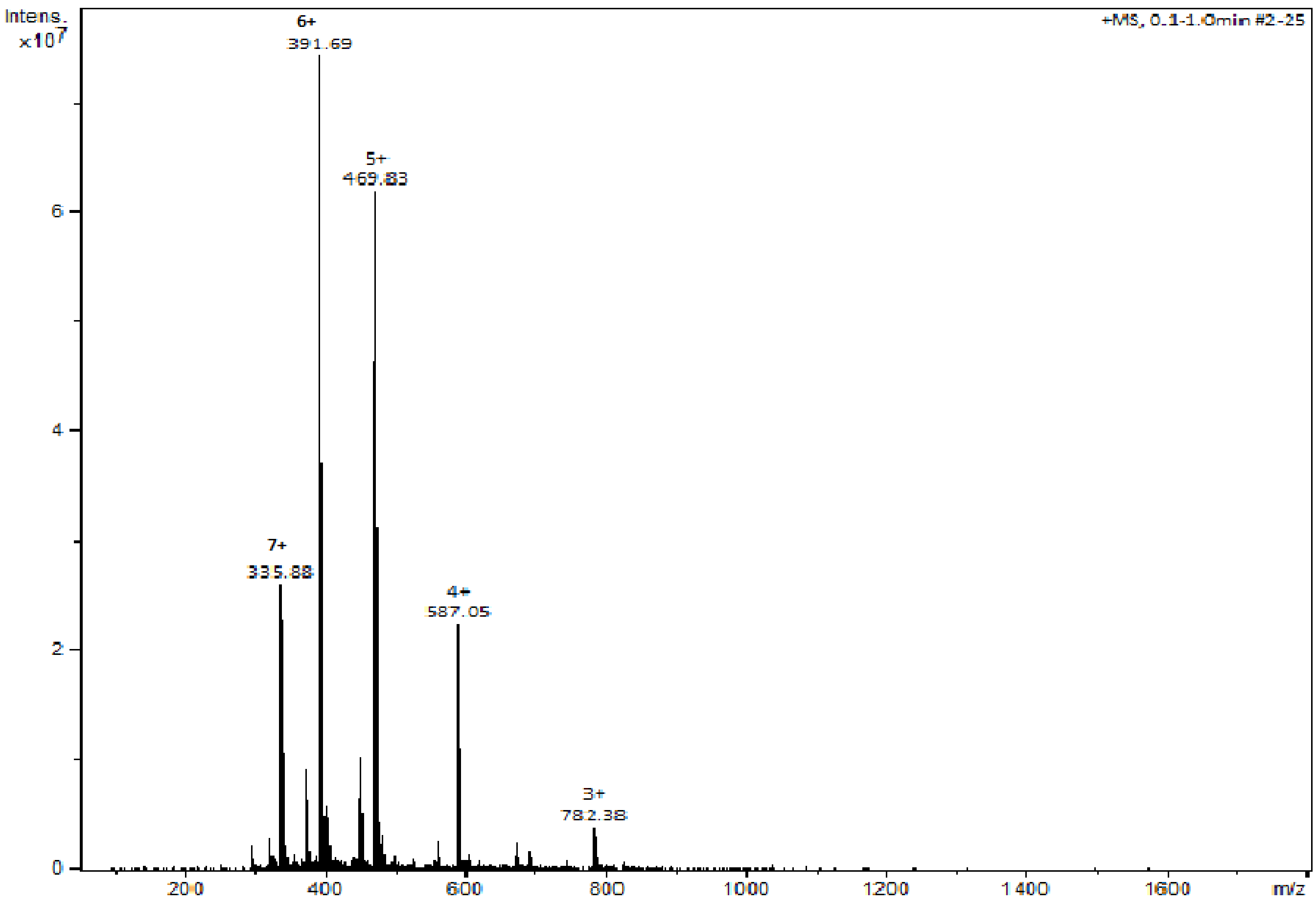

Figure S9. MS Spectrum of Cf-RQIKIWFQNRRK-azaGly-KK-*NH*$_2$. The identification of the conjugate was determined using Bruker Amazon SL (Germany). The sample is dissolved in water-acetonitrile (50:50) with 0.1% formic acid.

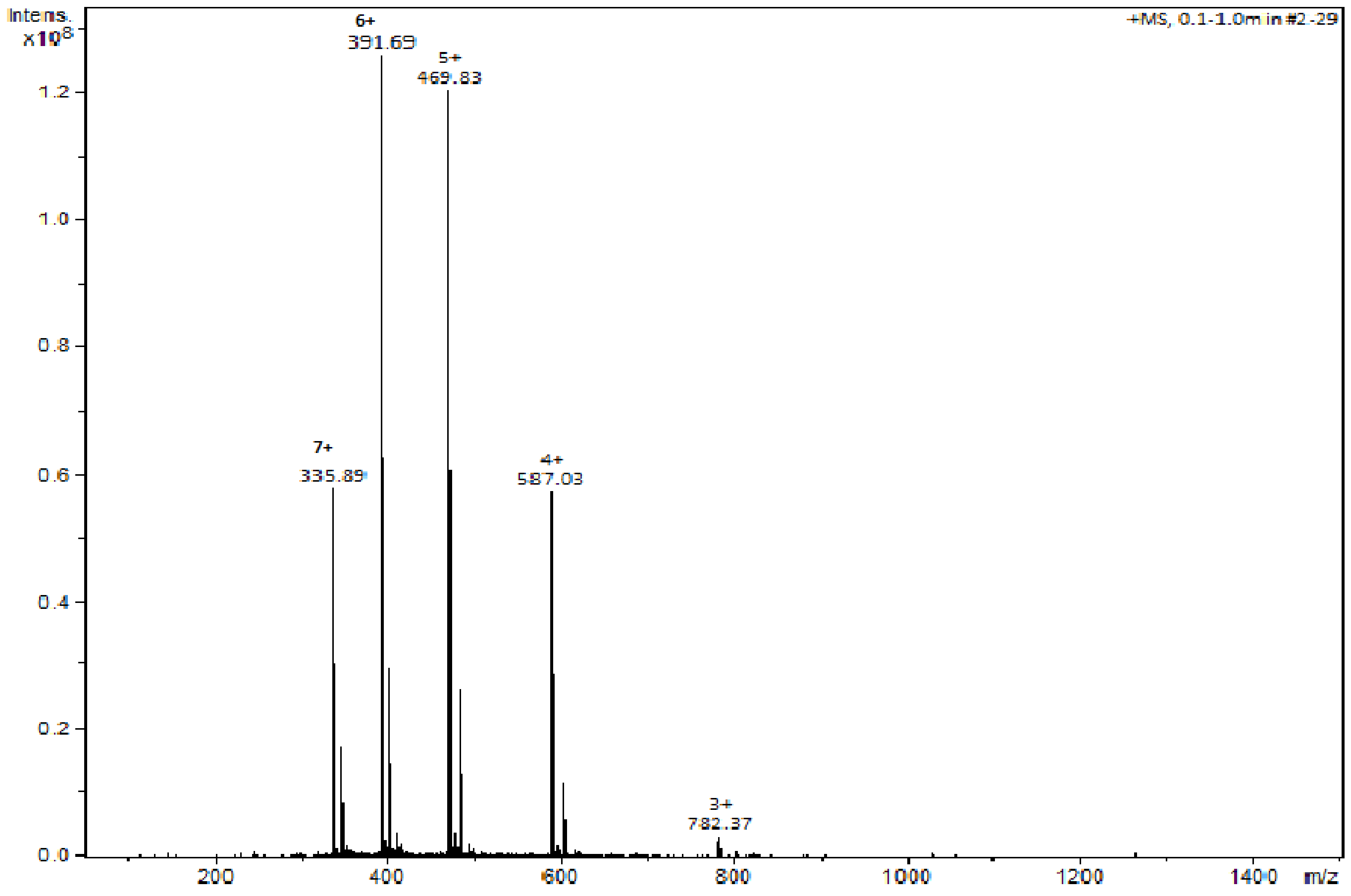

Figure S10. MS Spectrum of Cf-RQIKI-azaGly-FQNRRKWKK-*NH*$_2$. The identification of the conjugate was determined using Bruker Amazon SL (Germany). The sample is dissolved in water-acetonitrile (50:50) with 0.1% formic acid.

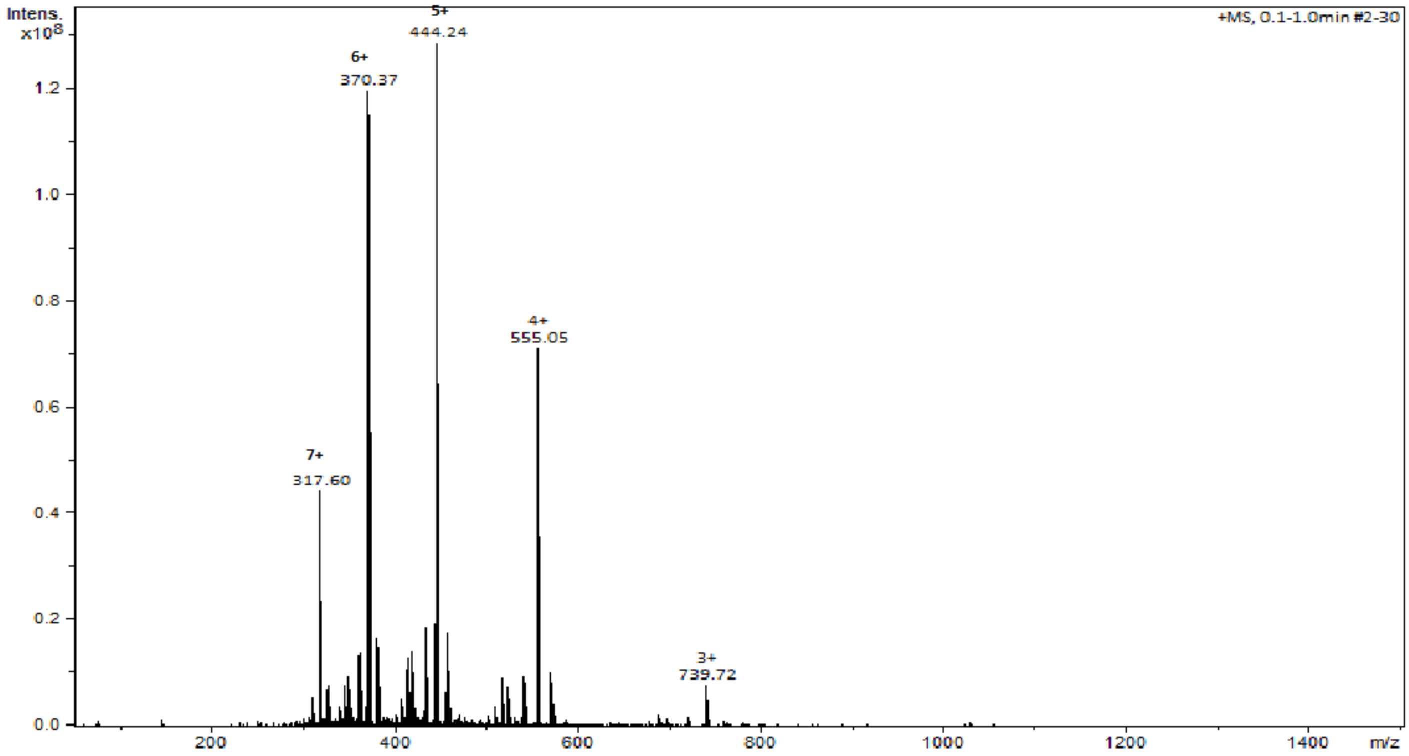

Figure S11. MS Spectrum of Cf-RQIKI-azaGly-FQNRRK-azaGly-KK-*NH*2. The identification of the conjugate was determined using Bruker Amazon SL (Germany). The sample is dissolved in water-acetonitrile (50:50) with 0.1% formic acid.

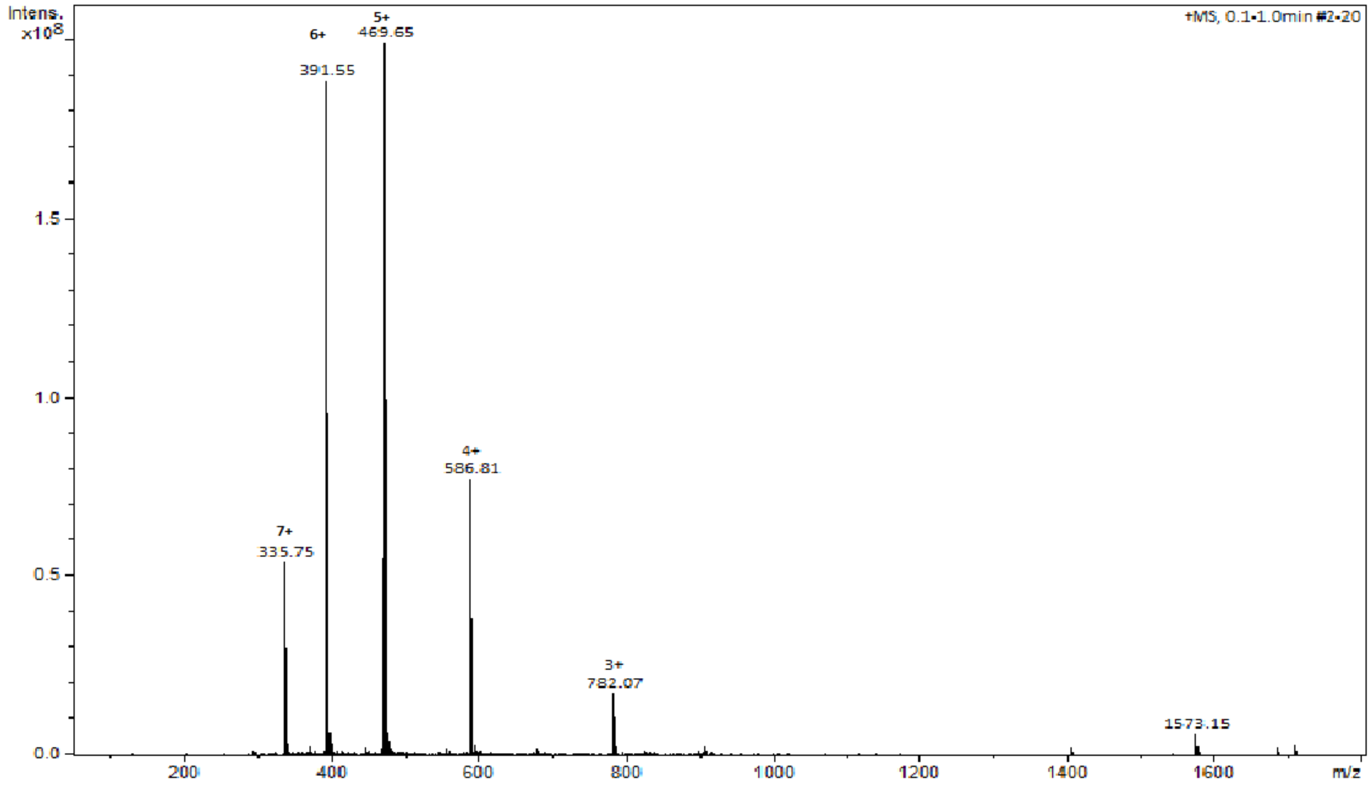

Figure S12. MS Spectrum of Cf-RQIKIWFQNRRKGKK-*NH*2. The identification of the conjugate was determined using Bruker Amazon SL (Germany). The sample is dissolved in water-acetonitrile (50:50) with 0.1% formic acid.

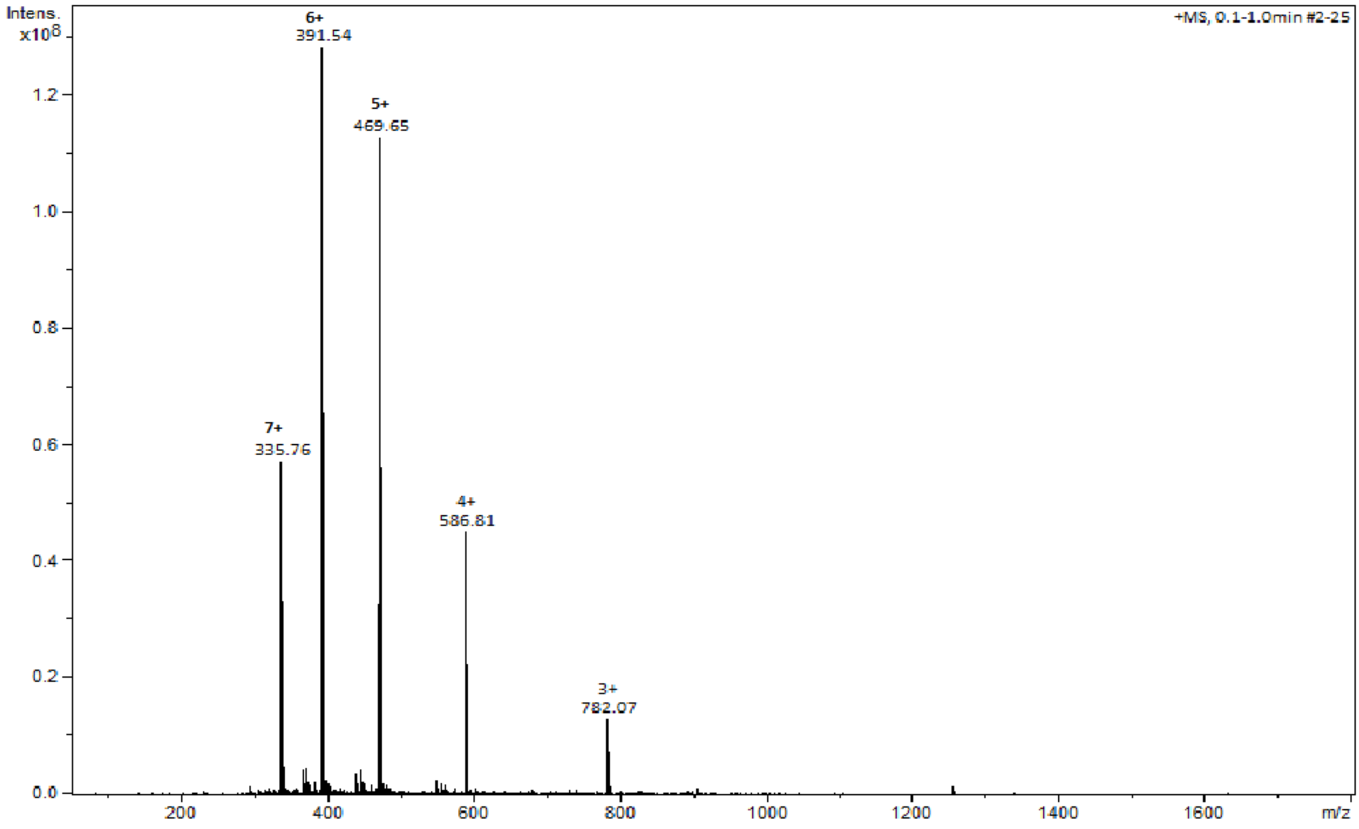

Figure S13. MS Spectrum of Cf-RQIKIGFQNRRKWKK-*NH2*. The identification of the conjugate was determined using Bruker Amazon SL (Germany). The sample is dissolved in water-acetonitrile (50:50) with 0.1% formic acid.

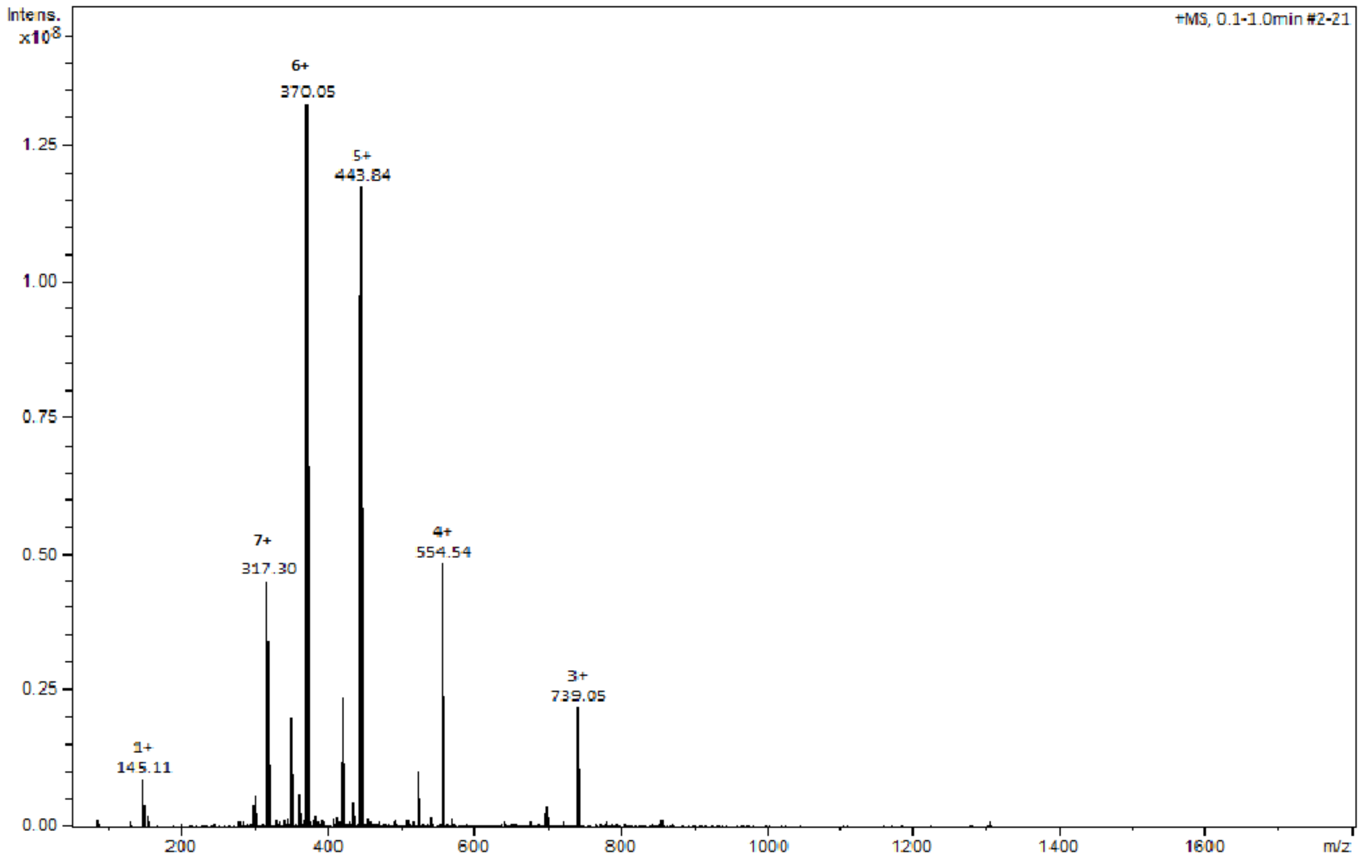

Figure S14. MS Spectrum of Cf-RQIKIGFQNRRKGKK-*NH2*. The identification of the conjugate was determined using Bruker Amazon SL (Germany). The sample is dissolved in water-acetonitrile (50:50) with 0.1% formic acid.

3. Cytotoxicity of Peptide Conjugates

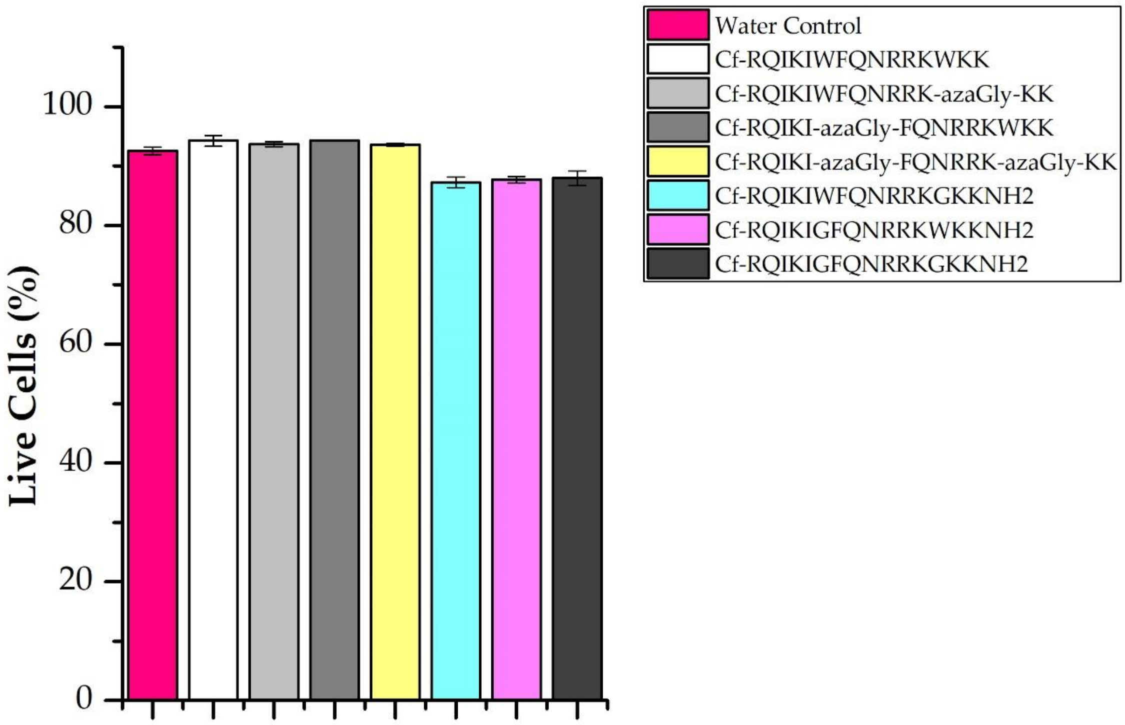

Figure S15. Evaluation of the cytotoxicity of peptides by examining the percentage of live cells by flow cytometry technique. Cells were incubated with 5 μM peptide solution for 90 min at 37°C.

4. ECD measurement

The UV-CD measurements were performed on a JASCO (Tokyo, Japan) J-1500 spectropolarimeter. Each spectrum was the average of five scans collected in the far UV (185-260 nm) range with a 0.1 mm path length quartz cell. The following settings were used throughout the measurements: a temperature control system at room temperature, the bandwidth of 1 nm, 0.2 nm step size, 4 s response time, and a scan rate of 50 nm/min. All spectra were corrected by subtracting the solvent spectrum acquired under identical conditions. All CD data were processed from mDeg to mean residue ellipticity (deg cm^2 dmol^{-1}) using the Spectra Analysis function of Jasco Spectra Manager, to account for the concentration differences.

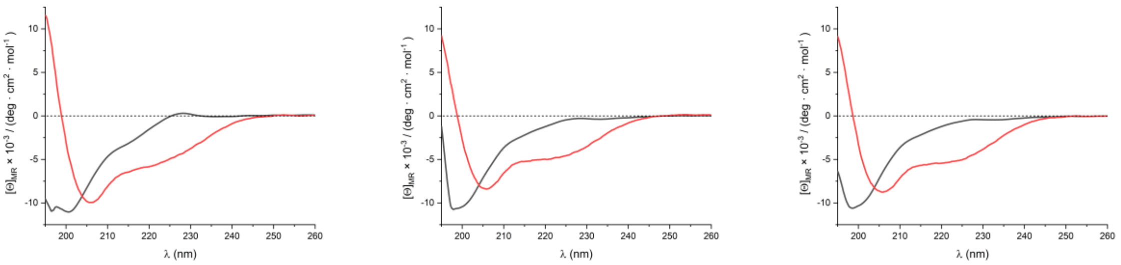

Figure S16. ECD spectra in PBS buffer, pH: 7.3 at 25°C (black) and 50 % TFE in PBS buffer (red) of Pen(desMet) (left panel), Trp56aGlyPen(desMet) (middle panel) and Trp56GlyPen(desMet) (right panel).

5. Calculations

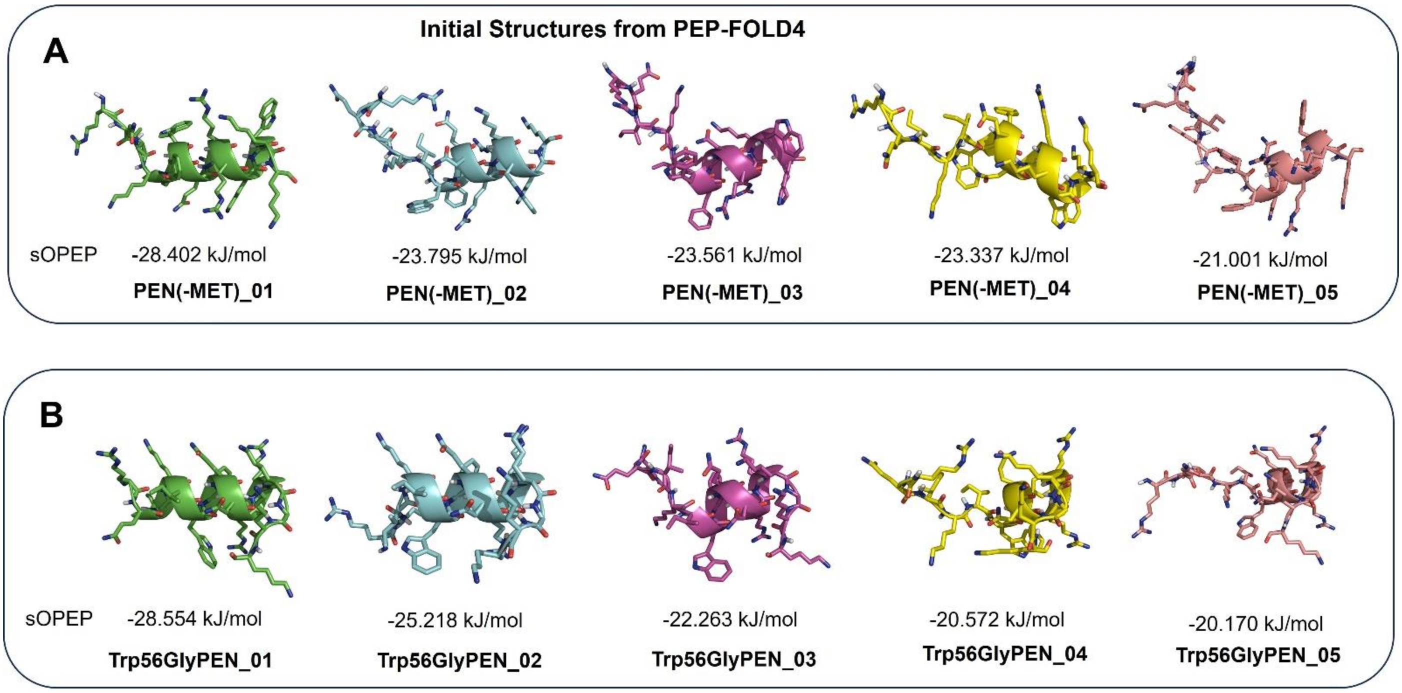

Figure S17. Initial structures of PEN(-Met) and Trp56GlyPen peptide were generated from PEP-Fold4. The best five structures of model peptides were shown with the sOPEP energy for each conformer.

Table S1. The coordinates of optimized structures of model peptides, Pen(-Met), Trp56aGlyPen, and Trp56GlyPen at the wB97XD/3-21G* level of theory.

```
TITLE     Pen(-Met)
REMARK   Created by GaussView 6.1.1, E = =-7066.4308708 a.u.
ATOM      1  N   ARG A   1     -14.562   0.239   1.661                       N
ATOM      2  CA  ARG A   1     -14.639  -0.989   0.840                       C
ATOM      3  C   ARG A   1     -13.569  -0.912  -0.271                       C
ATOM      4  O   ARG A   1     -13.555  -1.626  -1.287                       O
ATOM      5  CB  ARG A   1     -16.046  -1.261   0.263                       C
ATOM      6  CG  ARG A   1     -16.234  -2.701  -0.257                       C
ATOM      7  CD  ARG A   1     -17.695  -2.917  -0.692                       C
ATOM      8  NE  ARG A   1     -17.912  -4.317  -1.117                       N
ATOM      9  CZ  ARG A   1     -19.115  -4.903  -1.173                       C
ATOM     10  NH1 ARG A   1     -19.209  -6.225  -1.355                       N
ATOM     11  NH2 ARG A   1     -20.227  -4.174  -1.038                       N
ATOM     12  H   ARG A   1     -14.722   0.070   2.652                       H
ATOM     13  H01 ARG A   1     -21.130  -4.610  -0.898                       H
ATOM     14  H02 ARG A   1     -15.186   0.974   1.323                       H
ATOM     15  H03 ARG A   1     -14.356  -1.832   1.481                       H
ATOM     16  H04 ARG A   1     -16.253  -0.544  -0.541                       H
ATOM     17  H05 ARG A   1     -15.999  -3.411   0.545                       H
ATOM     18  H06 ARG A   1     -17.942  -2.233  -1.513                       H
ATOM     19  H07 ARG A   1     -17.099  -4.842  -1.418                       H
ATOM     20  H08 ARG A   1     -18.407  -6.836  -1.261                       H
ATOM     21  H09 ARG A   1     -20.216  -3.165  -1.112                       H
ATOM     22  H10 ARG A   1     -16.764  -1.077   1.071                       H
ATOM     23  H11 ARG A   1     -15.542  -2.861  -1.087                       H
ATOM     24  H12 ARG A   1     -18.357  -2.713   0.157                       H
ATOM     25  H13 ARG A   1     -20.093  -6.668  -1.579                       H
ATOM     26  N   GLN A   2     -12.621   0.021   0.014                       N
ATOM     27  CA  GLN A   2     -11.499   0.260  -0.882                       C
ATOM     28  C   GLN A   2     -10.285   0.679  -0.046                       C
ATOM     29  O   GLN A   2      -9.866   1.867  -0.044                       O
ATOM     30  CB  GLN A   2     -11.757   1.344  -1.935                       C
ATOM     31  CG  GLN A   2     -12.893   1.005  -2.903                       C
ATOM     32  CD  GLN A   2     -12.808   1.884  -4.142                       C
ATOM     33  NE2 GLN A   2     -13.616   1.562  -5.153                       N
ATOM     34  OE1 GLN A   2     -12.019   2.862  -4.229                       O
ATOM     35  H   GLN A   2     -12.859   0.590   0.838                       H
ATOM     36  H01 GLN A   2     -14.237   0.764  -5.120                       H
ATOM     37  H02 GLN A   2     -11.296  -0.692  -1.389                       H
ATOM     38  H03 GLN A   2     -10.835   1.476  -2.510                       H
ATOM     39  H04 GLN A   2     -13.863   1.175  -2.423                       H
ATOM     40  H05 GLN A   2     -13.612   2.128  -5.995                       H
ATOM     41  H06 GLN A   2     -11.966   2.286  -1.421                       H
ATOM     42  H07 GLN A   2     -12.866  -0.052  -3.183                       H
ATOM     43  N   ILE A   3      -9.679  -0.281   0.678                       N
ATOM     44  CA  ILE A   3      -8.604   0.045   1.640                       C
ATOM     45  C   ILE A   3      -7.276   0.209   0.866                       C
ATOM     46  O   ILE A   3      -6.331  -0.606   0.886                       O
ATOM     47  CB  ILE A   3      -8.501  -0.984   2.775                       C
ATOM     48  CG1 ILE A   3      -9.865  -1.150   3.483                       C
ATOM     49  CG2 ILE A   3      -7.431  -0.502   3.776                       C
ATOM     50  CD  ILE A   3      -9.860  -2.286   4.522                       C
ATOM     51  H   ILE A   3     -10.018  -1.236   0.641                       H
ATOM     52  H01 ILE A   3      -9.544  -3.230   4.064                       H
ATOM     53  H02 ILE A   3      -8.859   1.020   2.072                       H
ATOM     54  H03 ILE A   3      -8.193  -1.946   2.348                       H
ATOM     55  H04 ILE A   3     -10.649  -1.357   2.746                       H
ATOM     56  H05 ILE A   3      -7.309  -1.228   4.583                       H
ATOM     57  H06 ILE A   3     -10.863  -2.429   4.932                       H
ATOM     58  H07 ILE A   3     -10.125  -0.202   3.970                       H
ATOM     59  H08 ILE A   3      -7.737   0.451   4.225                       H
ATOM     60  H09 ILE A   3      -9.196  -2.062   5.361                       H
ATOM     61  H10 ILE A   3      -6.460  -0.383   3.290                       H
```

```
ATOM 62 N LYS A 4 -7.227 1.383 0.184 N
ATOM 63 CA LYS A 4 -6.141 1.709 -0.737 C
ATOM 64 C LYS A 4 -4.862 2.108 0.013 C
ATOM 65 O LYS A 4 -3.769 2.283 -0.582 O
ATOM 66 CB LYS A 4 -6.624 2.796 -1.718 C
ATOM 67 CG LYS A 4 -7.720 2.227 -2.651 C
ATOM 68 CD LYS A 4 -8.590 3.289 -3.356 C
ATOM 69 CE LYS A 4 -9.426 4.091 -2.343 C
ATOM 70 NZ LYS A 4 -10.640 4.692 -3.047 N
ATOM 71 H LYS A 4 -8.115 1.907 0.127 H
ATOM 72 H01 LYS A 4 -11.253 3.888 -3.466 H
ATOM 73 H02 LYS A 4 -5.864 0.809 -1.301 H
ATOM 74 H03 LYS A 4 -5.769 3.147 -2.302 H
ATOM 75 H04 LYS A 4 -8.391 1.589 -2.069 H
ATOM 76 H05 LYS A 4 -9.278 2.766 -4.032 H
ATOM 77 H06 LYS A 4 -8.862 4.914 -1.901 H
ATOM 78 H07 LYS A 4 -10.350 5.299 -3.827 H
ATOM 79 H08 LYS A 4 -7.003 3.640 -1.129 H
ATOM 80 H09 LYS A 4 -7.243 1.599 -3.411 H
ATOM 81 H10 LYS A 4 -7.970 3.959 -3.962 H
ATOM 82 H11 LYS A 4 -9.807 3.437 -1.554 H
ATOM 83 H12 LYS A 4 -11.208 5.253 -2.399 H
ATOM 84 N ILE A 5 -4.976 2.136 1.347 N
ATOM 85 CA ILE A 5 -3.799 2.135 2.207 C
ATOM 86 C ILE A 5 -2.878 1.003 1.767 C
ATOM 87 O ILE A 5 -1.634 1.113 1.868 O
ATOM 88 CB ILE A 5 -4.189 1.953 3.698 C
ATOM 89 CG1 ILE A 5 -5.242 3.005 4.115 C
ATOM 90 CG2 ILE A 5 -2.924 2.089 4.568 C
ATOM 91 CD ILE A 5 -5.691 2.852 5.579 C
ATOM 92 H ILE A 5 -5.898 2.052 1.754 H
ATOM 93 H01 ILE A 5 -6.033 1.829 5.772 H
ATOM 94 H02 ILE A 5 -3.245 3.068 2.109 H
ATOM 95 H03 ILE A 5 -4.617 0.949 3.814 H
ATOM 96 H04 ILE A 5 -6.136 2.925 3.484 H
ATOM 97 H05 ILE A 5 -2.557 3.120 4.524 H
ATOM 98 H06 ILE A 5 -6.516 3.535 5.797 H
ATOM 99 H07 ILE A 5 -4.817 4.003 3.950 H
ATOM 100 H08 ILE A 5 -2.111 1.442 4.233 H
ATOM 101 H09 ILE A 5 -4.880 3.082 6.274 H
ATOM 102 H10 ILE A 5 -3.155 1.845 5.607 H
ATOM 103 N TRP A 6 -3.445 -0.121 1.304 N
ATOM 104 CA TRP A 6 -2.565 -1.237 0.965 C
ATOM 105 C TRP A 6 -1.622 -0.904 -0.185 C
ATOM 106 O TRP A 6 -0.469 -1.390 -0.219 O
ATOM 107 CB TRP A 6 -3.413 -2.497 0.672 C
ATOM 108 CG TRP A 6 -3.848 -3.068 1.983 C
ATOM 109 CD1 TRP A 6 -5.063 -2.977 2.603 C
ATOM 110 CD2 TRP A 6 -2.947 -3.707 2.908 C
ATOM 111 CE2 TRP A 6 -3.685 -3.981 4.086 C
ATOM 112 CE3 TRP A 6 -1.589 -4.054 2.834 C
ATOM 113 NE1 TRP A 6 -4.975 -3.529 3.874 N
ATOM 114 CZ2 TRP A 6 -3.087 -4.592 5.192 C
ATOM 115 CZ3 TRP A 6 -0.998 -4.655 3.937 C
ATOM 116 CH2 TRP A 6 -1.743 -4.917 5.105 C
ATOM 117 H TRP A 6 -4.461 -0.255 1.245 H
ATOM 118 H01 TRP A 6 -1.260 -5.400 5.945 H
ATOM 119 H02 TRP A 6 -1.924 -1.443 1.823 H
ATOM 120 H03 TRP A 6 -2.791 -3.220 0.133 H
ATOM 121 H04 TRP A 6 -5.966 -2.529 2.225 H
ATOM 122 H05 TRP A 6 -1.025 -3.881 1.922 H
ATOM 123 H06 TRP A 6 -5.747 -3.654 4.513 H
ATOM 124 H07 TRP A 6 -3.651 -4.816 6.088 H
ATOM 125 H08 TRP A 6 0.043 -4.956 3.899 H
ATOM 126 H09 TRP A 6 -4.265 -2.231 0.039 H
ATOM 127 N PHE A 7 -2.099 -0.098 -1.148 N
```

ATOM	128	CA	PHE	A	7	-1.214	0.374	-2.218	C
ATOM	129	C	PHE	A	7	-0.083	1.194	-1.596	C
ATOM	130	O	PHE	A	7	1.119	1.028	-1.863	O
ATOM	131	CB	PHE	A	7	-2.028	1.248	-3.199	C
ATOM	132	CG	PHE	A	7	-1.112	2.002	-4.138	C
ATOM	133	CD1	PHE	A	7	-0.618	1.390	-5.291	C
ATOM	134	CD2	PHE	A	7	-0.721	3.309	-3.840	C
ATOM	135	CE1	PHE	A	7	0.250	2.075	-6.137	C
ATOM	136	CE2	PHE	A	7	0.146	3.996	-4.685	C
ATOM	137	CZ	PHE	A	7	0.633	3.380	-5.836	C
ATOM	138	H	PHE	A	7	-2.981	0.392	-1.014	H
ATOM	139	H01	PHE	A	7	1.287	3.920	-6.509	H
ATOM	140	H02	PHE	A	7	-0.750	-0.469	-2.731	H
ATOM	141	H03	PHE	A	7	-2.639	1.944	-2.616	H
ATOM	142	H04	PHE	A	7	-0.928	0.381	-5.539	H
ATOM	143	H05	PHE	A	7	-1.108	3.791	-2.948	H
ATOM	144	H06	PHE	A	7	0.610	1.600	-7.041	H
ATOM	145	H07	PHE	A	7	0.419	5.021	-4.462	H
ATOM	146	H08	PHE	A	7	-2.701	0.595	-3.764	H
ATOM	147	N	GLN	A	8	-0.508	2.115	-0.698	N
ATOM	148	CA	GLN	A	8	0.494	2.893	0.016	C
ATOM	149	C	GLN	A	8	1.533	1.933	0.621	C
ATOM	150	O	GLN	A	8	2.761	2.183	0.621	O
ATOM	151	CB	GLN	A	8	-0.102	3.633	1.220	C
ATOM	152	CG	GLN	A	8	-1.283	4.575	1.001	C
ATOM	153	CD	GLN	A	8	-1.726	5.059	2.388	C
ATOM	154	NE2	GLN	A	8	-2.833	5.822	2.417	N
ATOM	155	OE1	GLN	A	8	-1.113	4.749	3.435	O
ATOM	156	H	GLN	A	8	-1.492	2.189	-0.457	H
ATOM	157	H01	GLN	A	8	-3.300	6.126	1.574	H
ATOM	158	H02	GLN	A	8	1.025	3.564	-0.664	H
ATOM	159	H03	GLN	A	8	0.705	4.215	1.667	H
ATOM	160	H04	GLN	A	8	-2.123	4.068	0.511	H
ATOM	161	H05	GLN	A	8	-3.134	6.217	3.302	H
ATOM	162	H06	GLN	A	8	-0.433	2.876	1.932	H
ATOM	163	H07	GLN	A	8	-1.008	5.442	0.389	H
ATOM	164	N	ASN	A	9	0.988	0.882	1.243	N
ATOM	165	CA	ASN	A	9	1.808	-0.068	1.973	C
ATOM	166	C	ASN	A	9	2.812	-0.747	1.059	C
ATOM	167	O	ASN	A	9	3.991	-0.910	1.443	O
ATOM	168	CB	ASN	A	9	0.915	-1.115	2.638	C
ATOM	169	CG	ASN	A	9	0.067	-0.496	3.742	C
ATOM	170	ND2	ASN	A	9	-0.871	-1.301	4.251	N
ATOM	171	OD1	ASN	A	9	0.247	0.673	4.154	O
ATOM	172	H	ASN	A	9	-0.032	0.801	1.322	H
ATOM	173	H01	ASN	A	9	-0.994	-2.269	3.948	H
ATOM	174	H02	ASN	A	9	2.402	0.440	2.730	H
ATOM	175	H03	ASN	A	9	1.542	-1.897	3.081	H
ATOM	176	H04	ASN	A	9	-1.440	-0.972	5.022	H
ATOM	177	H05	ASN	A	9	0.284	-1.572	1.868	H
ATOM	178	N	ARG	A	10	2.378	-1.178	-0.139	N
ATOM	179	CA	ARG	A	10	3.406	-1.784	-0.978	C
ATOM	180	C	ARG	A	10	4.399	-0.724	-1.461	C
ATOM	181	O	ARG	A	10	5.592	-1.026	-1.706	O
ATOM	182	CB	ARG	A	10	2.909	-2.662	-2.144	C
ATOM	183	CG	ARG	A	10	4.120	-3.513	-2.587	C
ATOM	184	CD	ARG	A	10	3.878	-4.459	-3.766	C
ATOM	185	NE	ARG	A	10	5.169	-5.119	-4.057	N
ATOM	186	CZ	ARG	A	10	5.302	-6.288	-4.687	C
ATOM	187	NH1	ARG	A	10	6.493	-6.894	-4.703	N
ATOM	188	NH2	ARG	A	10	4.250	-6.859	-5.291	N
ATOM	189	H	ARG	A	10	1.393	-1.112	-0.414	H
ATOM	190	H01	ARG	A	10	4.298	-7.803	-5.653	H
ATOM	191	H02	ARG	A	10	4.000	-2.425	-0.323	H
ATOM	192	H03	ARG	A	10	2.530	-2.039	-2.961	H
ATOM	193	H04	ARG	A	10	4.464	-4.113	-1.737	H

ATOM	194	H05	ARG	A	10	3.526	-3.904	-4.644	H
ATOM	195	H06	ARG	A	10	6.013	-4.649	-3.720	H
ATOM	196	H07	ARG	A	10	7.272	-6.520	-4.161	H
ATOM	197	H08	ARG	A	10	3.391	-6.352	-5.448	H
ATOM	198	H09	ARG	A	10	2.098	-3.303	-1.781	H
ATOM	199	H10	ARG	A	10	4.934	-2.831	-2.841	H
ATOM	200	H11	ARG	A	10	3.140	-5.222	-3.495	H
ATOM	201	H12	ARG	A	10	6.667	-7.707	-5.281	H
ATOM	202	N	ARG	A	11	3.974	0.546	-1.585	N
ATOM	203	CA	ARG	A	11	5.019	1.534	-1.855	C
ATOM	204	C	ARG	A	11	6.044	1.525	-0.700	C
ATOM	205	O	ARG	A	11	7.278	1.550	-0.909	O
ATOM	206	CB	ARG	A	11	4.436	2.948	-2.034	C
ATOM	207	CG	ARG	A	11	5.556	3.886	-2.531	C
ATOM	208	CD	ARG	A	11	5.111	5.354	-2.607	C
ATOM	209	NE	ARG	A	11	6.234	6.173	-3.124	N
ATOM	210	CZ	ARG	A	11	6.112	7.409	-3.620	C
ATOM	211	NH1	ARG	A	11	7.137	7.982	-4.259	N
ATOM	212	NH2	ARG	A	11	4.959	8.075	-3.486	N
ATOM	213	H	ARG	A	11	2.987	0.809	-1.525	H
ATOM	214	H01	ARG	A	11	4.788	8.937	-3.990	H
ATOM	215	H02	ARG	A	11	5.586	1.239	-2.742	H
ATOM	216	H03	ARG	A	11	4.013	3.270	-1.078	H
ATOM	217	H04	ARG	A	11	5.868	3.563	-3.530	H
ATOM	218	H05	ARG	A	11	4.818	5.705	-1.610	H
ATOM	219	H06	ARG	A	11	7.159	5.760	-3.069	H
ATOM	220	H07	ARG	A	11	7.950	7.453	-4.547	H
ATOM	221	H08	ARG	A	11	4.244	7.768	-2.841	H
ATOM	222	H09	ARG	A	11	3.625	2.905	-2.771	H
ATOM	223	H10	ARG	A	11	6.422	3.796	-1.864	H
ATOM	224	H11	ARG	A	11	4.264	5.450	-3.295	H
ATOM	225	H12	ARG	A	11	7.136	8.970	-4.487	H
ATOM	226	N	LYS	A	12	5.511	1.541	0.533	N
ATOM	227	CA	LYS	A	12	6.378	1.563	1.719	C
ATOM	228	C	LYS	A	12	7.358	0.374	1.704	C
ATOM	229	O	LYS	A	12	8.555	0.521	2.017	O
ATOM	230	CB	LYS	A	12	5.501	1.502	2.991	C
ATOM	231	CG	LYS	A	12	4.637	2.771	3.156	C
ATOM	232	CD	LYS	A	12	3.456	2.577	4.145	C
ATOM	233	CE	LYS	A	12	2.334	3.556	3.775	C
ATOM	234	NZ	LYS	A	12	0.996	3.211	4.416	N
ATOM	235	H	LYS	A	12	4.494	1.540	0.652	H
ATOM	236	H01	LYS	A	12	0.749	2.191	4.238	H
ATOM	237	H02	LYS	A	12	7.005	2.458	1.718	H
ATOM	238	H03	LYS	A	12	6.153	1.368	3.860	H
ATOM	239	H04	LYS	A	12	4.215	3.022	2.180	H
ATOM	240	H05	LYS	A	12	3.781	2.710	5.181	H
ATOM	241	H06	LYS	A	12	2.176	3.491	2.700	H
ATOM	242	H07	LYS	A	12	0.221	3.812	4.019	H
ATOM	243	H08	LYS	A	12	4.861	0.620	2.894	H
ATOM	244	H09	LYS	A	12	5.263	3.609	3.481	H
ATOM	245	H10	LYS	A	12	3.067	1.560	4.043	H
ATOM	246	H11	LYS	A	12	2.581	4.585	4.046	H
ATOM	247	H12	LYS	A	12	1.032	3.352	5.433	H
ATOM	248	N	TRP	A	13	6.784	-0.788	1.341	N
ATOM	249	CA	TRP	A	13	7.494	-2.060	1.211	C
ATOM	250	C	TRP	A	13	8.759	-1.954	0.382	C
ATOM	251	O	TRP	A	13	9.683	-2.773	0.485	O
ATOM	252	CB	TRP	A	13	6.518	-3.046	0.497	C
ATOM	253	CG	TRP	A	13	7.193	-4.243	-0.096	C
ATOM	254	CD1	TRP	A	13	7.537	-5.422	0.507	C
ATOM	255	CD2	TRP	A	13	7.713	-4.305	-1.442	C
ATOM	256	CE2	TRP	A	13	8.369	-5.560	-1.583	C
ATOM	257	CE3	TRP	A	13	7.694	-3.402	-2.516	C
ATOM	258	NE1	TRP	A	13	8.238	-6.224	-0.387	N
ATOM	259	CZ2	TRP	A	13	9.009	-5.918	-2.776	C

```
ATOM    260  CZ3 TRP A  13       8.316  -3.768  -3.706                       C
ATOM    261  CH2 TRP A  13       8.976  -5.011  -3.831                       C
ATOM    262  H   TRP A  13       5.771  -0.810   1.195                       H
ATOM    263  H01 TRP A  13       9.475  -5.258  -4.762                       H
ATOM    264  H02 TRP A  13       7.808  -2.459   2.179                       H
ATOM    265  H03 TRP A  13       6.076  -2.468  -0.316                       H
ATOM    266  H04 TRP A  13       7.356  -5.754   1.516                       H
ATOM    267  H05 TRP A  13       7.219  -2.434  -2.401                       H
ATOM    268  H06 TRP A  13       8.623  -7.131  -0.166                       H
ATOM    269  H07 TRP A  13       9.540  -6.858  -2.868                       H
ATOM    270  H08 TRP A  13       8.311  -3.086  -4.549                       H
ATOM    271  H09 TRP A  13       5.732  -3.329   1.204                       H
ATOM    272  N   LYS A  14       8.822  -0.938  -0.520                       N
ATOM    273  CA  LYS A  14      10.066  -0.860  -1.257                       C
ATOM    274  C   LYS A  14      11.242  -0.759  -0.278                       C
ATOM    275  O   LYS A  14      12.393  -1.200  -0.589                       O
ATOM    276  CB  LYS A  14      10.070   0.309  -2.277                       C
ATOM    277  CG  LYS A  14      10.416  -0.158  -3.704                       C
ATOM    278  CD  LYS A  14      11.825  -0.802  -3.829                       C
ATOM    279  CE  LYS A  14      11.830  -2.348  -3.685                       C
ATOM    280  NZ  LYS A  14      12.769  -2.778  -2.563                       N
ATOM    281  H   LYS A  14       8.110  -0.217  -0.629                       H
ATOM    282  H01 LYS A  14      12.618  -3.760  -2.303                       H
ATOM    283  H02 LYS A  14      10.207  -1.816  -1.765                       H
ATOM    284  H03 LYS A  14      10.793   1.070  -1.963                       H
ATOM    285  H04 LYS A  14      10.373   0.710  -4.369                       H
ATOM    286  H05 LYS A  14      12.476  -0.364  -3.065                       H
ATOM    287  H06 LYS A  14      12.162  -2.837  -4.603                       H
ATOM    288  H07 LYS A  14      12.584  -2.114  -1.667                       H
ATOM    289  H08 LYS A  14       9.085   0.785  -2.265                       H
ATOM    290  H09 LYS A  14       9.653  -0.865  -4.052                       H
ATOM    291  H10 LYS A  14      12.254  -0.544  -4.801                       H
ATOM    292  H11 LYS A  14      10.843  -2.735  -3.426                       H
ATOM    293  H12 LYS A  14      13.750  -2.661  -2.846                       H
ATOM    294  N   LYS A  15      11.030  -0.161   0.887                       N
ATOM    295  CA  LYS A  15      12.045  -0.263   1.949                       C
ATOM    296  C   LYS A  15      11.304  -0.271   3.281                       C
ATOM    297  O   LYS A  15      11.488   0.506   4.205                       O
ATOM    298  CB  LYS A  15      13.088   0.861   1.905                       C
ATOM    299  CG  LYS A  15      14.225   0.584   2.913                       C
ATOM    300  CD  LYS A  15      15.308   1.688   2.907                       C
ATOM    301  CE  LYS A  15      16.355   1.355   3.976                       C
ATOM    302  NZ  LYS A  15      17.462   2.436   4.040                       N
ATOM    303  H   LYS A  15      10.076   0.118   1.173                       H
ATOM    304  H01 LYS A  15      18.162   2.203   4.763                       H
ATOM    305  H02 LYS A  15      12.539  -1.233   1.824                       H
ATOM    306  H04 LYS A  15      12.600   1.810   2.144                       H
ATOM    307  H05 LYS A  15      14.690  -0.381   2.677                       H
ATOM    308  H06 LYS A  15      14.836   2.653   3.129                       H
ATOM    309  H07 LYS A  15      16.865   0.413   3.763                       H
ATOM    310  H08 LYS A  15      17.054   3.356   4.268                       H
ATOM    311  H09 LYS A  15      13.490   0.902   0.888                       H
ATOM    312  H10 LYS A  15      13.779   0.526   3.909                       H
ATOM    313  H11 LYS A  15      15.777   1.743   1.917                       H
ATOM    314  H12 LYS A  15      15.910   1.318   4.972                       H
ATOM    315  H13 LYS A  15      17.947   2.512   3.132                       H
TER     316      LYS A  15
HETATM  317  O           0      10.411  -1.294   3.318                       O
HETATM  318  H           0       9.908  -1.265   4.168                       H
END
CONECT  296  317
CONECT  317  296  318
CONECT  318  317
```

```
TITLE       Trp56aGlyPen
REMARK   Created by GaussView 6.1.1: E= -6682.6460495 a.u.
ATOM      1  N   ARG A   1     -10.525  -1.430  -2.573                       N
ATOM      2  CA  ARG A   1     -11.220  -1.197  -1.266                       C
ATOM      3  C   ARG A   1     -10.042  -1.186  -0.283                       C
ATOM      4  O   ARG A   1      -9.519  -0.127   0.120                       O
ATOM      5  CB  ARG A   1     -12.331  -2.238  -1.046                       C
ATOM      6  CG  ARG A   1     -13.181  -2.052   0.228                       C
ATOM      7  CD  ARG A   1     -14.356  -3.053   0.196                       C
ATOM      8  NE  ARG A   1     -15.240  -2.893   1.369                       N
ATOM      9  CZ  ARG A   1     -16.506  -3.343   1.413                       C
ATOM     10  NH1 ARG A   1     -17.307  -2.984   2.421                       N
ATOM     11  NH2 ARG A   1     -16.971  -4.141   0.449                       N
ATOM     12  H   ARG A   1     -10.766  -0.738  -3.282                       H
ATOM     13  H01 ARG A   1     -17.961  -4.346   0.367                       H
ATOM     14  H02 ARG A   1     -10.681  -2.372  -2.938                       H
ATOM     15  H03 ARG A   1     -11.639  -0.189  -1.260                       H
ATOM     16  H04 ARG A   1     -11.905  -3.250  -1.052                       H
ATOM     17  H05 ARG A   1     -13.581  -1.033   0.264                       H
ATOM     18  H06 ARG A   1     -13.963  -4.077   0.158                       H
ATOM     19  H07 ARG A   1     -14.844  -2.481   2.206                       H
ATOM     20  H08 ARG A   1     -17.067  -2.234   3.056                       H
ATOM     21  H09 ARG A   1     -16.357  -4.619  -0.198                       H
ATOM     22  H10 ARG A   1     -12.989  -2.172  -1.921                       H
ATOM     23  H11 ARG A   1     -12.564  -2.222   1.119                       H
ATOM     24  H12 ARG A   1     -14.952  -2.868  -0.704                       H
ATOM     25  H13 ARG A   1     -18.196  -3.444   2.580                       H
ATOM     26  N   GLN A   2      -9.470  -2.403  -0.100                       N
ATOM     27  CA  GLN A   2      -8.097  -2.526   0.370                       C
ATOM     28  C   GLN A   2      -7.107  -1.890  -0.608                       C
ATOM     29  O   GLN A   2      -5.896  -1.824  -0.263                       O
ATOM     30  CB  GLN A   2      -7.680  -3.980   0.603                       C
ATOM     31  CG  GLN A   2      -8.381  -4.678   1.776                       C
ATOM     32  CD  GLN A   2      -7.381  -5.669   2.404                       C
ATOM     33  NE2 GLN A   2      -7.923  -6.743   3.008                       N
ATOM     34  OE1 GLN A   2      -6.161  -5.439   2.367                       O
ATOM     35  H   GLN A   2      -9.908  -3.238  -0.467                       H
ATOM     36  H01 GLN A   2      -8.915  -6.934   3.011                       H
ATOM     37  H02 GLN A   2      -7.984  -1.966   1.305                       H
ATOM     38  H03 GLN A   2      -6.613  -3.978   0.846                       H
ATOM     39  H04 GLN A   2      -9.303  -5.183   1.478                       H
ATOM     40  H05 GLN A   2      -7.311  -7.406   3.468                       H
ATOM     41  H06 GLN A   2      -7.805  -4.555  -0.322                       H
ATOM     42  H07 GLN A   2      -8.624  -3.945   2.557                       H
ATOM     43  N   ILE A   3      -7.554  -1.350  -1.747                       N
ATOM     44  CA  ILE A   3      -6.615  -0.549  -2.535                       C
ATOM     45  C   ILE A   3      -5.973   0.525  -1.664                       C
ATOM     46  O   ILE A   3      -4.840   0.970  -1.936                       O
ATOM     47  CB  ILE A   3      -7.300   0.116  -3.754                       C
ATOM     48  CG1 ILE A   3      -8.381   1.128  -3.304                       C
ATOM     49  CG2 ILE A   3      -7.825  -0.967  -4.714                       C
ATOM     50  CD  ILE A   3      -9.210   1.682  -4.475                       C
ATOM     51  H   ILE A   3      -8.534  -1.439  -2.067                       H
ATOM     52  H01 ILE A   3      -9.877   2.479  -4.133                       H
ATOM     53  H02 ILE A   3      -5.795  -1.177  -2.892                       H
ATOM     54  H03 ILE A   3      -6.506   0.672  -4.268                       H
ATOM     55  H04 ILE A   3      -7.886   1.962  -2.797                       H
ATOM     56  H05 ILE A   3      -7.041  -1.697  -4.936                       H
ATOM     57  H06 ILE A   3      -9.824   0.904  -4.940                       H
ATOM     58  H07 ILE A   3      -9.041   0.656  -2.568                       H
ATOM     59  H08 ILE A   3      -8.138  -0.521  -5.661                       H
ATOM     60  H09 ILE A   3      -8.559   2.099  -5.250                       H
ATOM     61  H10 ILE A   3      -8.679  -1.494  -4.282                       H
ATOM     62  N   LYS A   4      -6.691   1.006  -0.620                       N
ATOM     63  CA  LYS A   4      -6.067   2.041   0.200                       C
ATOM     64  C   LYS A   4      -4.848   1.486   0.956                       C
```

ATOM 65 O LYS A 4 -3.850 2.208 1.198 O
ATOM 66 CB LYS A 4 -7.079 2.642 1.189 C
ATOM 67 CG LYS A 4 -8.272 3.310 0.461 C
ATOM 68 CD LYS A 4 -8.948 4.371 1.364 C
ATOM 69 CE LYS A 4 -10.065 5.091 0.601 C
ATOM 70 NZ LYS A 4 -10.663 6.241 1.449 N
ATOM 71 H LYS A 4 -7.626 0.651 -0.402 H
ATOM 72 H01 LYS A 4 -9.935 6.936 1.681 H
ATOM 73 H02 LYS A 4 -5.673 2.828 -0.449 H
ATOM 74 H03 LYS A 4 -6.537 3.386 1.783 H
ATOM 75 H04 LYS A 4 -8.996 2.540 0.169 H
ATOM 76 H05 LYS A 4 -8.188 5.099 1.674 H
ATOM 77 H06 LYS A 4 -10.896 4.424 0.363 H
ATOM 78 H07 LYS A 4 -11.054 5.879 2.334 H
ATOM 79 H08 LYS A 4 -7.438 1.859 1.866 H
ATOM 80 H09 LYS A 4 -7.906 3.800 -0.449 H
ATOM 81 H10 LYS A 4 -9.351 3.888 2.262 H
ATOM 82 H11 LYS A 4 -9.691 5.558 -0.313 H
ATOM 83 H12 LYS A 4 -11.418 6.727 0.936 H
ATOM 84 N ILE A 5 -4.952 0.214 1.363 N
ATOM 85 CA ILE A 5 -3.810 -0.466 1.974 C
ATOM 86 C ILE A 5 -2.743 -0.689 0.912 C
ATOM 87 O ILE A 5 -1.522 -0.584 1.174 O
ATOM 88 CB ILE A 5 -4.227 -1.805 2.626 C
ATOM 89 CG1 ILE A 5 -5.377 -1.550 3.624 C
ATOM 90 CG2 ILE A 5 -3.004 -2.419 3.333 C
ATOM 91 CD ILE A 5 -5.803 -2.827 4.368 C
ATOM 92 H ILE A 5 -5.673 -0.393 0.976 H
ATOM 93 H01 ILE A 5 -5.921 -3.661 3.668 H
ATOM 94 H02 ILE A 5 -3.378 0.172 2.743 H
ATOM 95 H03 ILE A 5 -4.587 -2.473 1.836 H
ATOM 96 H04 ILE A 5 -6.240 -1.146 3.079 H
ATOM 97 H05 ILE A 5 -3.245 -3.423 3.690 H
ATOM 98 H06 ILE A 5 -6.749 -2.666 4.897 H
ATOM 99 H07 ILE A 5 -5.054 -0.779 4.336 H
ATOM 100 H08 ILE A 5 -2.719 -1.796 4.188 H
ATOM 101 H09 ILE A 5 -5.056 -3.116 5.112 H
ATOM 102 H10 ILE A 5 -2.133 -2.484 2.675 H
ATOM 103 N TRP A 6 -3.175 -1.016 -0.311 N
ATOM 104 CA TRP A 6 -2.184 -1.196 -1.368 C
ATOM 105 C TRP A 6 -1.370 0.090 -1.548 C
ATOM 106 O TRP A 6 -0.129 0.081 -1.698 O
ATOM 107 CB TRP A 6 -2.880 -1.585 -2.692 C
ATOM 108 CG TRP A 6 -1.870 -2.172 -3.622 C
ATOM 109 CD1 TRP A 6 -1.332 -1.645 -4.765 C
ATOM 110 CD2 TRP A 6 -1.202 -3.430 -3.386 C
ATOM 111 CE2 TRP A 6 -0.256 -3.602 -4.426 C
ATOM 112 CE3 TRP A 6 -1.352 -4.426 -2.410 C
ATOM 113 NE1 TRP A 6 -0.356 -2.502 -5.256 N
ATOM 114 CZ2 TRP A 6 0.561 -4.734 -4.487 C
ATOM 115 CZ3 TRP A 6 -0.536 -5.547 -2.470 C
ATOM 116 CH2 TRP A 6 0.418 -5.692 -3.498 C
ATOM 117 H TRP A 6 -4.160 -1.235 -0.486 H
ATOM 118 H01 TRP A 6 1.028 -6.585 -3.530 H
ATOM 119 H02 TRP A 6 -1.478 -1.975 -1.077 H
ATOM 120 H03 TRP A 6 -3.381 -0.704 -3.103 H
ATOM 121 H04 TRP A 6 -1.573 -0.726 -5.273 H
ATOM 122 H05 TRP A 6 -2.122 -4.341 -1.650 H
ATOM 123 H06 TRP A 6 0.144 -2.380 -6.124 H
ATOM 124 H07 TRP A 6 1.274 -4.871 -5.290 H
ATOM 125 H08 TRP A 6 -0.656 -6.345 -1.747 H
ATOM 126 H09 TRP A 6 -3.648 -2.336 -2.460 H
ATOM 127 N PHE A 7 -2.089 1.226 -1.527 N
ATOM 128 CA PHE A 7 -1.413 2.522 -1.572 C
ATOM 129 C PHE A 7 -0.387 2.611 -0.438 C
ATOM 130 O PHE A 7 0.797 2.958 -0.622 O

ATOM	131	CB	PHE	A	7	-2.474	3.635	-1.410	C
ATOM	132	CG	PHE	A	7	-1.824	4.997	-1.331	C
ATOM	133	CD1	PHE	A	7	-1.552	5.727	-2.490	C
ATOM	134	CD2	PHE	A	7	-1.452	5.526	-0.092	C
ATOM	135	CE1	PHE	A	7	-0.925	6.967	-2.413	C
ATOM	136	CE2	PHE	A	7	-0.820	6.764	-0.014	C
ATOM	137	CZ	PHE	A	7	-0.556	7.488	-1.175	C
ATOM	138	H	PHE	A	7	-3.114	1.195	-1.524	H
ATOM	139	H01	PHE	A	7	-0.085	8.462	-1.115	H
ATOM	140	H02	PHE	A	7	-0.854	2.653	-2.501	H
ATOM	141	H03	PHE	A	7	-3.046	3.428	-0.500	H
ATOM	142	H04	PHE	A	7	-1.853	5.331	-3.454	H
ATOM	143	H05	PHE	A	7	-1.668	4.966	0.810	H
ATOM	144	H06	PHE	A	7	-0.742	7.539	-3.315	H
ATOM	145	H07	PHE	A	7	-0.553	7.175	0.953	H
ATOM	146	H08	PHE	A	7	-3.159	3.580	-2.263	H
ATOM	147	N	GLN	A	8	-0.871	2.285	0.780	N
ATOM	148	CA	GLN	A	8	0.037	2.272	1.920	C
ATOM	149	C	GLN	A	8	1.276	1.431	1.586	C
ATOM	150	O	GLN	A	8	2.426	1.766	1.964	O
ATOM	151	CB	GLN	A	8	-0.568	1.551	3.132	C
ATOM	152	CG	GLN	A	8	-1.825	2.103	3.796	C
ATOM	153	CD	GLN	A	8	-2.226	1.091	4.872	C
ATOM	154	NE2	GLN	A	8	-3.405	1.316	5.482	N
ATOM	155	OE1	GLN	A	8	-1.521	0.097	5.164	O
ATOM	156	H	GLN	A	8	-1.854	2.038	0.905	H
ATOM	157	H01	GLN	A	8	-3.958	2.139	5.290	H
ATOM	158	H02	GLN	A	8	0.380	3.280	2.166	H
ATOM	159	H03	GLN	A	8	0.210	1.525	3.895	H
ATOM	160	H04	GLN	A	8	-2.642	2.221	3.075	H
ATOM	161	H05	GLN	A	8	-3.692	0.705	6.238	H
ATOM	162	H06	GLN	A	8	-0.788	0.531	2.813	H
ATOM	163	H07	GLN	A	8	-1.640	3.075	4.270	H
ATOM	164	N	ASN	A	9	1.013	0.268	0.990	N
ATOM	165	CA	ASN	A	9	2.083	-0.665	0.675	C
ATOM	166	C	ASN	A	9	3.111	-0.008	-0.235	C
ATOM	167	O	ASN	A	9	4.333	-0.133	-0.010	O
ATOM	168	CB	ASN	A	9	1.492	-1.907	0.012	C
ATOM	169	CG	ASN	A	9	0.634	-2.694	0.990	C
ATOM	170	ND2	ASN	A	9	-0.063	-3.698	0.436	N
ATOM	171	OD1	ASN	A	9	0.589	-2.453	2.216	O
ATOM	172	H	ASN	A	9	0.043	-0.022	0.816	H
ATOM	173	H01	ASN	A	9	-0.014	-3.930	-0.554	H
ATOM	174	H02	ASN	A	9	2.626	-0.947	1.575	H
ATOM	175	H03	ASN	A	9	2.302	-2.555	-0.340	H
ATOM	176	H04	ASN	A	9	-0.621	-4.294	1.037	H
ATOM	177	H05	ASN	A	9	0.893	-1.589	-0.848	H
ATOM	178	N	ARG	A	10	2.629	0.699	-1.268	N
ATOM	179	CA	ARG	A	10	3.588	1.418	-2.100	C
ATOM	180	C	ARG	A	10	4.381	2.431	-1.260	C
ATOM	181	O	ARG	A	10	5.585	2.674	-1.468	O
ATOM	182	CB	ARG	A	10	2.875	2.172	-3.242	C
ATOM	183	CG	ARG	A	10	3.941	2.716	-4.216	C
ATOM	184	CD	ARG	A	10	3.369	3.691	-5.256	C
ATOM	185	NE	ARG	A	10	4.469	4.136	-6.144	N
ATOM	186	CZ	ARG	A	10	4.299	4.730	-7.329	C
ATOM	187	NH1	ARG	A	10	5.341	4.908	-8.149	N
ATOM	188	NH2	ARG	A	10	3.083	5.143	-7.705	N
ATOM	189	H	ARG	A	10	1.620	0.742	-1.450	H
ATOM	190	H01	ARG	A	10	2.899	5.448	-8.653	H
ATOM	191	H02	ARG	A	10	4.329	0.724	-2.506	H
ATOM	192	H03	ARG	A	10	2.264	2.966	-2.797	H
ATOM	193	H04	ARG	A	10	4.400	1.872	-4.741	H
ATOM	194	H05	ARG	A	10	2.921	4.554	-4.749	H
ATOM	195	H06	ARG	A	10	5.413	3.987	-5.805	H
ATOM	196	H07	ARG	A	10	6.232	4.455	-7.985	H

ATOM	197	H08	ARG	A	10	2.319	5.203	-7.046	H
ATOM	198	H09	ARG	A	10	2.208	1.476	-3.762	H
ATOM	199	H10	ARG	A	10	4.727	3.213	-3.637	H
ATOM	200	H11	ARG	A	10	2.610	3.188	-5.865	H
ATOM	201	H12	ARG	A	10	5.278	5.499	-8.969	H
ATOM	202	N	ARG	A	11	3.657	3.090	-0.328	N
ATOM	203	CA	ARG	A	11	4.347	4.052	0.517	C
ATOM	204	C	ARG	A	11	5.431	3.385	1.373	C
ATOM	205	O	ARG	A	11	6.470	4.007	1.709	O
ATOM	206	CB	ARG	A	11	3.335	4.763	1.441	C
ATOM	207	CG	ARG	A	11	4.011	5.792	2.366	C
ATOM	208	CD	ARG	A	11	4.625	6.956	1.567	C
ATOM	209	NE	ARG	A	11	5.521	7.797	2.377	N
ATOM	210	CZ	ARG	A	11	6.850	7.613	2.433	C
ATOM	211	NH1	ARG	A	11	7.611	8.589	2.961	N
ATOM	212	NH2	ARG	A	11	7.410	6.505	1.959	N
ATOM	213	H	ARG	A	11	2.645	2.965	-0.246	H
ATOM	214	H01	ARG	A	11	8.413	6.487	1.816	H
ATOM	215	H02	ARG	A	11	4.885	4.761	-0.117	H
ATOM	216	H03	ARG	A	11	2.852	3.981	2.032	H
ATOM	217	H04	ARG	A	11	4.799	5.306	2.946	H
ATOM	218	H05	ARG	A	11	3.837	7.598	1.164	H
ATOM	219	H06	ARG	A	11	5.109	8.541	2.927	H
ATOM	220	H07	ARG	A	11	7.251	9.524	3.107	H
ATOM	221	H08	ARG	A	11	6.918	5.590	1.797	H
ATOM	222	H09	ARG	A	11	2.570	5.243	0.819	H
ATOM	223	H10	ARG	A	11	3.274	6.187	3.073	H
ATOM	224	H11	ARG	A	11	5.213	6.583	0.727	H
ATOM	225	H12	ARG	A	11	8.578	8.429	3.207	H
ATOM	226	N	LYS	A	12	5.172	2.143	1.789	N
ATOM	227	CA	LYS	A	12	6.174	1.356	2.519	C
ATOM	228	C	LYS	A	12	7.374	1.096	1.586	C
ATOM	229	O	LYS	A	12	8.543	1.321	1.927	O
ATOM	230	CB	LYS	A	12	5.520	0.043	3.001	C
ATOM	231	CG	LYS	A	12	4.423	0.332	4.051	C
ATOM	232	CD	LYS	A	12	3.403	-0.827	4.224	C
ATOM	233	CE	LYS	A	12	2.064	-0.224	4.673	C
ATOM	234	NZ	LYS	A	12	0.880	-1.172	4.537	N
ATOM	235	H	LYS	A	12	4.255	1.735	1.594	H
ATOM	236	H01	LYS	A	12	-0.025	-0.664	4.750	H
ATOM	237	H02	LYS	A	12	6.565	1.937	3.358	H
ATOM	238	H03	LYS	A	12	6.291	-0.615	3.413	H
ATOM	239	H04	LYS	A	12	3.871	1.213	3.712	H
ATOM	240	H05	LYS	A	12	3.767	-1.577	4.932	H
ATOM	241	H06	LYS	A	12	1.851	0.618	4.019	H
ATOM	242	H07	LYS	A	12	0.968	-1.961	5.190	H
ATOM	243	H08	LYS	A	12	5.079	-0.443	2.125	H
ATOM	244	H09	LYS	A	12	4.882	0.574	5.015	H
ATOM	245	H10	LYS	A	12	3.253	-1.318	3.259	H
ATOM	246	H11	LYS	A	12	2.099	0.119	5.709	H
ATOM	247	H12	LYS	A	12	0.818	-1.577	3.554	H
ATOM	248	N	GLY	A	13	6.985	0.597	0.378	N
ATOM	249	C	GLY	A	13	8.925	-0.090	-0.977	C
ATOM	250	O	GLY	A	13	9.812	0.261	-1.770	O
ATOM	251	H	GLY	A	13	6.001	0.411	0.166	H
ATOM	252	H01	GLY	A	13	7.732	1.574	-1.260	H
ATOM	253	N	LYS	A	14	8.961	-1.268	-0.284	N
ATOM	254	CA	LYS	A	14	10.156	-2.083	-0.375	C
ATOM	255	C	LYS	A	14	9.744	-3.543	-0.215	C
ATOM	256	O	LYS	A	14	8.587	-3.890	0.045	O
ATOM	257	CB	LYS	A	14	11.163	-1.756	0.773	C
ATOM	258	CG	LYS	A	14	11.243	-0.248	1.030	C
ATOM	259	CD	LYS	A	14	12.088	0.067	2.280	C
ATOM	260	CE	LYS	A	14	11.833	1.522	2.669	C
ATOM	261	NZ	LYS	A	14	12.612	1.862	3.967	N
ATOM	262	H	LYS	A	14	8.157	-1.664	0.185	H

```
ATOM    263  H01 LYS A  14      12.309   1.237   4.730                 H
ATOM    264  H02 LYS A  14      10.623  -1.887  -1.344                 H
ATOM    265  H03 LYS A  14      12.155  -2.137   0.502                 H
ATOM    266  H04 LYS A  14      10.231   0.128   1.200                 H
ATOM    267  H05 LYS A  14      13.153  -0.108   2.085                 H
ATOM    268  H06 LYS A  14      10.770   1.693   2.858                 H
ATOM    269  H07 LYS A  14      12.462   2.839   4.258                 H
ATOM    270  H08 LYS A  14      10.844  -2.286   1.676                 H
ATOM    271  H09 LYS A  14      11.641   0.262   0.147                 H
ATOM    272  H10 LYS A  14      11.767  -0.594   3.095                 H
ATOM    273  H11 LYS A  14      12.197   2.214   1.906                 H
ATOM    274  H12 LYS A  14      13.623   1.715   3.817                 H
ATOM    275  N   LYS A  15      10.776  -4.444  -0.292                 N
ATOM    276  CA  LYS A  15      10.593  -5.758   0.302                 C
ATOM    277  O   LYS A  15      10.542  -4.558   2.454                 O
ATOM    278  CB  LYS A  15      11.766  -6.708   0.000                 C
ATOM    279  CG  LYS A  15      11.887  -7.091  -1.486                 C
ATOM    280  CD  LYS A  15      13.036  -8.113  -1.671                 C
ATOM    281  CE  LYS A  15      13.146  -8.556  -3.133                 C
ATOM    282  NZ  LYS A  15      14.274  -9.606  -3.296                 N
ATOM    283  H   LYS A  15      11.723  -4.107  -0.407                 H
ATOM    284  H01 LYS A  15      15.181  -9.210  -3.002                 H
ATOM    285  H02 LYS A  15       9.653  -6.186  -0.056                 H
ATOM    286  H04 LYS A  15      11.605  -7.607   0.600                 H
ATOM    287  H05 LYS A  15      12.073  -6.198  -2.093                 H
ATOM    288  H06 LYS A  15      12.836  -8.983  -1.033                 H
ATOM    289  H07 LYS A  15      13.404  -7.727  -3.794                 H
ATOM    290  H08 LYS A  15      14.357  -9.913  -4.279                 H
ATOM    291  H09 LYS A  15      12.700  -6.245   0.344                 H
ATOM    292  H10 LYS A  15      10.942  -7.535  -1.820                 H
ATOM    293  H11 LYS A  15      13.979  -7.658  -1.345                 H
ATOM    294  H12 LYS A  15      12.231  -9.039  -3.480                 H
ATOM    295  H13 LYS A  15      14.080 -10.435  -2.712                 H
TER     296      LYS A  15
HETATM  297  C          0      10.469  -5.598   1.820                 C
HETATM  298  N          0       7.826   0.711  -0.738                 N
HETATM  299  O          0      10.264  -6.820   2.381                 O
HETATM  300  H          0      10.186  -6.756   3.364                 H
END
CONECT  248  298
CONECT  249  298
CONECT  252  298
CONECT  276  297
CONECT  277  297
CONECT  297  276  277  299
CONECT  298  252  248  249
CONECT  299  297  300
CONECT  300  299
```

TITLE Trp56GlyPen

```
REMARK   Created by GaussView 6.1.1: E =  =-6666.7260931 a.u.
ATOM      1  N   ARG A   1      -9.850  -1.035  -2.673                 N
ATOM      2  CA  ARG A   1     -10.572  -0.635  -1.422                 C
ATOM      3  C   ARG A   1      -9.429  -0.604  -0.399                 C
ATOM      4  O   ARG A   1      -8.857   0.456  -0.070                 O
ATOM      5  CB  ARG A   1     -11.757  -1.577  -1.149                 C
ATOM      6  CG  ARG A   1     -12.650  -1.201   0.052                 C
ATOM      7  CD  ARG A   1     -13.893  -2.118   0.060                 C
ATOM      8  NE  ARG A   1     -14.815  -1.775   1.162                 N
ATOM      9  CZ  ARG A   1     -16.114  -2.123   1.182                 C
ATOM     10  NH1 ARG A   1     -16.929  -1.605   2.105                 N
ATOM     11  NH2 ARG A   1     -16.595  -2.978   0.277                 N
ATOM     12  H   ARG A   1     -10.030  -0.405  -3.455                 H
ATOM     13  H01 ARG A   1     -17.594  -3.115   0.161                 H
ATOM     14  H02 ARG A   1     -10.051  -1.998  -2.951                 H
```

ATOM	15	H03	ARG	A	1	-10.922	0.393	-1.527	H
ATOM	16	H04	ARG	A	1	-11.401	-2.608	-1.026	H
ATOM	17	H05	ARG	A	1	-12.976	-0.159	-0.038	H
ATOM	18	H06	ARG	A	1	-13.574	-3.164	0.147	H
ATOM	19	H07	ARG	A	1	-14.426	-1.314	1.977	H
ATOM	20	H08	ARG	A	1	-16.660	-0.813	2.676	H
ATOM	21	H09	ARG	A	1	-15.993	-3.566	-0.285	H
ATOM	22	H10	ARG	A	1	-12.371	-1.566	-2.058	H
ATOM	23	H11	ARG	A	1	-12.089	-1.316	0.987	H
ATOM	24	H12	ARG	A	1	-14.432	-1.987	-0.884	H
ATOM	25	H13	ARG	A	1	-17.858	-1.980	2.261	H
ATOM	26	N	GLN	A	2	-8.935	-1.830	-0.092	N
ATOM	27	CA	GLN	A	2	-7.587	-1.989	0.438	C
ATOM	28	C	GLN	A	2	-6.529	-1.503	-0.556	C
ATOM	29	O	GLN	A	2	-5.327	-1.473	-0.176	O
ATOM	30	CB	GLN	A	2	-7.272	-3.438	0.818	C
ATOM	31	CG	GLN	A	2	-8.064	-3.978	2.016	C
ATOM	32	CD	GLN	A	2	-7.170	-4.991	2.758	C
ATOM	33	NE2	GLN	A	2	-7.818	-5.962	3.429	N
ATOM	34	OE1	GLN	A	2	-5.934	-4.866	2.745	O
ATOM	35	H	GLN	A	2	-9.410	-2.668	-0.401	H
ATOM	36	H01	GLN	A	2	-8.822	-6.068	3.415	H
ATOM	37	H02	GLN	A	2	-7.471	-1.352	1.322	H
ATOM	38	H03	GLN	A	2	-6.218	-3.481	1.105	H
ATOM	39	H04	GLN	A	2	-9.015	-4.431	1.725	H
ATOM	40	H05	GLN	A	2	-7.278	-6.631	3.965	H
ATOM	41	H06	GLN	A	2	-7.400	-4.087	-0.056	H
ATOM	42	H07	GLN	A	2	-8.270	-3.166	2.726	H
ATOM	43	N	ILE	A	3	-6.909	-1.040	-1.752	N
ATOM	44	CA	ILE	A	3	-5.905	-0.348	-2.563	C
ATOM	45	C	ILE	A	3	-5.241	0.759	-1.751	C
ATOM	46	O	ILE	A	3	-4.075	1.115	-2.006	O
ATOM	47	CB	ILE	A	3	-6.513	0.248	-3.855	C
ATOM	48	CG1	ILE	A	3	-7.557	1.344	-3.528	C
ATOM	49	CG2	ILE	A	3	-7.062	-0.882	-4.746	C
ATOM	50	CD	ILE	A	3	-8.318	1.839	-4.770	C
ATOM	51	H	ILE	A	3	-7.882	-1.102	-2.098	H
ATOM	52	H01	ILE	A	3	-8.953	2.693	-4.518	H
ATOM	53	H02	ILE	A	3	-5.101	-1.038	-2.836	H
ATOM	54	H03	ILE	A	3	-5.676	0.722	-4.381	H
ATOM	55	H04	ILE	A	3	-7.037	2.194	-3.072	H
ATOM	56	H05	ILE	A	3	-6.309	-1.664	-4.881	H
ATOM	57	H06	ILE	A	3	-8.955	1.057	-5.193	H
ATOM	58	H07	ILE	A	3	-8.263	0.967	-2.781	H
ATOM	59	H08	ILE	A	3	-7.321	-0.498	-5.736	H
ATOM	60	H09	ILE	A	3	-7.621	2.159	-5.552	H
ATOM	61	H10	ILE	A	3	-7.955	-1.331	-4.305	H
ATOM	62	N	LYS	A	4	-5.974	1.363	-0.783	N
ATOM	63	CA	LYS	A	4	-5.327	2.429	-0.023	C
ATOM	64	C	LYS	A	4	-4.176	1.879	0.836	C
ATOM	65	O	LYS	A	4	-3.163	2.576	1.087	O
ATOM	66	CB	LYS	A	4	-6.342	3.169	0.865	C
ATOM	67	CG	LYS	A	4	-7.455	3.851	0.031	C
ATOM	68	CD	LYS	A	4	-8.101	5.016	0.823	C
ATOM	69	CE	LYS	A	4	-9.133	5.747	-0.044	C
ATOM	70	NZ	LYS	A	4	-9.692	6.990	0.692	N
ATOM	71	H	LYS	A	4	-6.935	1.077	-0.576	H
ATOM	72	H01	LYS	A	4	-8.931	7.654	0.911	H
ATOM	73	H02	LYS	A	4	-4.862	3.136	-0.716	H
ATOM	74	H03	LYS	A	4	-5.779	3.922	1.427	H
ATOM	75	H04	LYS	A	4	-8.212	3.107	-0.241	H
ATOM	76	H05	LYS	A	4	-7.310	5.716	1.119	H
ATOM	77	H06	LYS	A	4	-9.994	5.117	-0.276	H
ATOM	78	H07	LYS	A	4	-10.146	6.719	1.579	H
ATOM	79	H08	LYS	A	4	-6.782	2.466	1.580	H
ATOM	80	H09	LYS	A	4	-7.016	4.250	-0.892	H

ATOM	81	H10	LYS	A	4	-8.576	4.625	1.731		H
ATOM	82	H11	LYS	A	4	-8.688	6.123	-0.968		H
ATOM	83	H12	LYS	A	4	-10.389	7.484	0.109		H
ATOM	84	N	ILE	A	5	-4.355	0.643	1.319		N
ATOM	85	CA	ILE	A	5	-3.276	-0.031	2.040		C
ATOM	86	C	ILE	A	5	-2.162	-0.365	1.061		C
ATOM	87	O	ILE	A	5	-0.952	-0.259	1.377		O
ATOM	88	CB	ILE	A	5	-3.784	-1.302	2.756		C
ATOM	89	CG1	ILE	A	5	-4.924	-0.914	3.723		C
ATOM	90	CG2	ILE	A	5	-2.609	-1.957	3.503		C
ATOM	91	CD	ILE	A	5	-5.457	-2.115	4.524		C
ATOM	92	H	ILE	A	5	-5.077	0.035	0.934		H
ATOM	93	H01	ILE	A	5	-5.608	-2.982	3.872		H
ATOM	94	H02	ILE	A	5	-2.862	0.645	2.786		H
ATOM	95	H03	ILE	A	5	-4.181	-1.987	1.999		H
ATOM	96	H04	ILE	A	5	-5.744	-0.473	3.142		H
ATOM	97	H05	ILE	A	5	-2.940	-2.878	3.987		H
ATOM	98	H06	ILE	A	5	-6.406	-1.862	5.009		H
ATOM	99	H07	ILE	A	5	-4.553	-0.134	4.401		H
ATOM	100	H08	ILE	A	5	-2.216	-1.271	4.263		H
ATOM	101	H09	ILE	A	5	-4.756	-2.406	5.310		H
ATOM	102	H10	ILE	A	5	-1.791	-2.202	2.819		H
ATOM	103	N	TRP	A	6	-2.532	-0.791	-0.158		N
ATOM	104	CA	TRP	A	6	-1.442	-1.076	-1.084		C
ATOM	105	C	TRP	A	6	-0.658	0.206	-1.366		C
ATOM	106	O	TRP	A	6	0.587	0.198	-1.490		O
ATOM	107	CB	TRP	A	6	-1.877	-1.737	-2.421		C
ATOM	108	CG	TRP	A	6	-0.620	-2.389	-2.926		C
ATOM	109	CD1	TRP	A	6	0.424	-1.770	-3.559		C
ATOM	110	CD2	TRP	A	6	-0.082	-3.592	-2.331		C
ATOM	111	CE2	TRP	A	6	1.307	-3.614	-2.605		C
ATOM	112	CE3	TRP	A	6	-0.651	-4.624	-1.570		C
ATOM	113	NE1	TRP	A	6	1.587	-2.504	-3.378		N
ATOM	114	CZ2	TRP	A	6	2.148	-4.587	-2.062		C
ATOM	115	CZ3	TRP	A	6	0.173	-5.634	-1.094		C
ATOM	116	CH2	TRP	A	6	1.566	-5.598	-1.315		C
ATOM	117	H	TRP	A	6	-3.508	-0.997	-0.385		H
ATOM	118	H01	TRP	A	6	2.190	-6.368	-0.879		H
ATOM	119	H02	TRP	A	6	-0.744	-1.752	-0.588		H
ATOM	120	H03	TRP	A	6	-2.290	-0.981	-3.096		H
ATOM	121	H04	TRP	A	6	0.442	-0.827	-4.077		H
ATOM	122	H05	TRP	A	6	-1.716	-4.642	-1.371		H
ATOM	123	H06	TRP	A	6	2.491	-2.274	-3.760		H
ATOM	124	H07	TRP	A	6	3.224	-4.495	-2.134		H
ATOM	125	H08	TRP	A	6	-0.253	-6.453	-0.528		H
ATOM	126	H09	TRP	A	6	-2.651	-2.485	-2.217		H
ATOM	127	N	PHE	A	7	-1.378	1.331	-1.482		N
ATOM	128	CA	PHE	A	7	-0.692	2.615	-1.638		C
ATOM	129	C	PHE	A	7	0.329	2.788	-0.507		C
ATOM	130	O	PHE	A	7	1.513	3.133	-0.705		O
ATOM	131	CB	PHE	A	7	-1.747	3.742	-1.587		C
ATOM	132	CG	PHE	A	7	-1.098	5.102	-1.700		C
ATOM	133	CD1	PHE	A	7	-0.913	5.704	-2.946		C
ATOM	134	CD2	PHE	A	7	-0.648	5.761	-0.554		C
ATOM	135	CE1	PHE	A	7	-0.295	6.948	-3.045		C
ATOM	136	CE2	PHE	A	7	-0.025	7.002	-0.650		C
ATOM	137	CZ	PHE	A	7	0.152	7.599	-1.897		C
ATOM	138	H	PHE	A	7	-2.403	1.301	-1.528		H
ATOM	139	H01	PHE	A	7	0.615	8.575	-1.971		H
ATOM	140	H02	PHE	A	7	-0.125	2.659	-2.572		H
ATOM	141	H03	PHE	A	7	-2.296	3.649	-0.645		H
ATOM	142	H04	PHE	A	7	-1.276	5.207	-3.840		H
ATOM	143	H05	PHE	A	7	-0.797	5.299	0.417		H
ATOM	144	H06	PHE	A	7	-0.183	7.423	-4.012		H
ATOM	145	H07	PHE	A	7	0.305	7.513	0.246		H
ATOM	146	H08	PHE	A	7	-2.453	3.579	-2.409		H

ATOM	147	N	GLN	A	8	-0.156	2.532	0.726	N
ATOM	148	CA	GLN	A	8	0.753	2.565	1.866	C
ATOM	149	C	GLN	A	8	1.980	1.683	1.584	C
ATOM	150	O	GLN	A	8	3.139	2.049	1.895	O
ATOM	151	CB	GLN	A	8	0.121	1.953	3.124	C
ATOM	152	CG	GLN	A	8	-1.149	2.578	3.694	C
ATOM	153	CD	GLN	A	8	-1.624	1.667	4.828	C
ATOM	154	NE2	GLN	A	8	-2.824	1.969	5.357	N
ATOM	155	OE1	GLN	A	8	-0.961	0.686	5.236	O
ATOM	156	H	GLN	A	8	-1.138	2.291	0.860	H
ATOM	157	H01	GLN	A	8	-3.347	2.784	5.068	H
ATOM	158	H02	GLN	A	8	1.123	3.578	2.045	H
ATOM	159	H03	GLN	A	8	0.881	1.995	3.903	H
ATOM	160	H04	GLN	A	8	-1.937	2.654	2.935	H
ATOM	161	H05	GLN	A	8	-3.162	1.432	6.148	H
ATOM	162	H06	GLN	A	8	-0.104	0.912	2.895	H
ATOM	163	H07	GLN	A	8	-0.962	3.581	4.096	H
ATOM	164	N	ASN	A	9	1.707	0.475	1.084	N
ATOM	165	CA	ASN	A	9	2.784	-0.471	0.819	C
ATOM	166	C	ASN	A	9	3.765	0.073	-0.208	C
ATOM	167	O	ASN	A	9	4.998	-0.102	-0.073	O
ATOM	168	CB	ASN	A	9	2.206	-1.812	0.366	C
ATOM	169	CG	ASN	A	9	1.414	-2.422	1.512	C
ATOM	170	ND2	ASN	A	9	0.571	-3.407	1.186	N
ATOM	171	OD1	ASN	A	9	1.561	-2.029	2.693	O
ATOM	172	H	ASN	A	9	0.736	0.180	0.942	H
ATOM	173	H01	ASN	A	9	0.483	-3.782	0.245	H
ATOM	174	H02	ASN	A	9	3.369	-0.624	1.723	H
ATOM	175	H03	ASN	A	9	3.028	-2.483	0.077	H
ATOM	176	H04	ASN	A	9	0.053	-3.870	1.924	H
ATOM	177	H05	ASN	A	9	1.573	-1.648	-0.511	H
ATOM	178	N	ARG	A	10	3.244	0.773	-1.225	N
ATOM	179	CA	ARG	A	10	4.167	1.459	-2.121	C
ATOM	180	C	ARG	A	10	5.047	2.425	-1.318	C
ATOM	181	O	ARG	A	10	6.279	2.508	-1.513	O
ATOM	182	CB	ARG	A	10	3.413	2.246	-3.212	C
ATOM	183	CG	ARG	A	10	4.436	2.833	-4.206	C
ATOM	184	CD	ARG	A	10	3.809	3.817	-5.206	C
ATOM	185	NE	ARG	A	10	4.874	4.328	-6.102	N
ATOM	186	CZ	ARG	A	10	4.659	4.938	-7.273	C
ATOM	187	NH1	ARG	A	10	5.679	5.180	-8.103	N
ATOM	188	NH2	ARG	A	10	3.420	5.302	-7.622	N
ATOM	189	H	ARG	A	10	2.227	0.833	-1.351	H
ATOM	190	H01	ARG	A	10	3.208	5.615	-8.562	H
ATOM	191	H02	ARG	A	10	4.863	0.746	-2.572	H
ATOM	192	H03	ARG	A	10	2.811	3.018	-2.721	H
ATOM	193	H04	ARG	A	10	4.904	2.014	-4.764	H
ATOM	194	H05	ARG	A	10	3.339	4.647	-4.665	H
ATOM	195	H06	ARG	A	10	5.829	4.222	-5.779	H
ATOM	196	H07	ARG	A	10	6.593	4.766	-7.961	H
ATOM	197	H08	ARG	A	10	2.665	5.318	-6.951	H
ATOM	198	H09	ARG	A	10	2.731	1.564	-3.732	H
ATOM	199	H10	ARG	A	10	5.228	3.342	-3.645	H
ATOM	200	H11	ARG	A	10	3.057	3.304	-5.815	H
ATOM	201	H12	ARG	A	10	5.578	5.785	-8.909	H
ATOM	202	N	ARG	A	11	4.412	3.197	-0.415	N
ATOM	203	CA	ARG	A	11	5.216	4.116	0.388	C
ATOM	204	C	ARG	A	11	6.247	3.384	1.260	C
ATOM	205	O	ARG	A	11	7.310	3.941	1.619	O
ATOM	206	CB	ARG	A	11	4.297	4.967	1.293	C
ATOM	207	CG	ARG	A	11	5.079	5.921	2.218	C
ATOM	208	CD	ARG	A	11	5.934	6.929	1.427	C
ATOM	209	NE	ARG	A	11	6.814	7.730	2.295	N
ATOM	210	CZ	ARG	A	11	8.098	7.417	2.543	C
ATOM	211	NH1	ARG	A	11	8.877	8.326	3.151	N
ATOM	212	NH2	ARG	A	11	8.595	6.238	2.183	N

ATOM	213	H	ARG	A	11	3.396	3.153	-0.296	H
ATOM	214	H01	ARG	A	11	9.597	6.092	2.210	H
ATOM	215	H02	ARG	A	11	5.807	4.742	-0.285	H
ATOM	216	H03	ARG	A	11	3.711	4.262	1.890	H
ATOM	217	H04	ARG	A	11	5.734	5.348	2.879	H
ATOM	218	H05	ARG	A	11	5.293	7.619	0.872	H
ATOM	219	H06	ARG	A	11	6.421	8.551	2.738	H
ATOM	220	H07	ARG	A	11	8.596	9.294	3.244	H
ATOM	221	H08	ARG	A	11	8.020	5.412	1.909	H
ATOM	222	H09	ARG	A	11	3.610	5.533	0.654	H
ATOM	223	H10	ARG	A	11	4.370	6.468	2.847	H
ATOM	224	H11	ARG	A	11	6.573	6.412	0.709	H
ATOM	225	H12	ARG	A	11	9.792	8.084	3.508	H
ATOM	226	N	LYS	A	12	5.912	2.146	1.644	N
ATOM	227	CA	LYS	A	12	6.819	1.297	2.408	C
ATOM	228	C	LYS	A	12	7.907	0.653	1.536	C
ATOM	229	O	LYS	A	12	8.818	-0.014	2.077	O
ATOM	230	CB	LYS	A	12	6.021	0.140	3.054	C
ATOM	231	CG	LYS	A	12	4.976	0.641	4.067	C
ATOM	232	CD	LYS	A	12	3.932	-0.439	4.454	C
ATOM	233	CE	LYS	A	12	2.658	0.264	4.948	C
ATOM	234	NZ	LYS	A	12	1.436	-0.647	4.943	N
ATOM	235	H	LYS	A	12	4.965	1.815	1.454	H
ATOM	236	H01	LYS	A	12	0.540	-0.087	5.041	H
ATOM	237	H02	LYS	A	12	7.326	1.896	3.170	H
ATOM	238	H03	LYS	A	12	6.743	-0.538	3.518	H
ATOM	239	H04	LYS	A	12	4.439	1.469	3.598	H
ATOM	240	H05	LYS	A	12	4.327	-1.124	5.211	H
ATOM	241	H06	LYS	A	12	2.433	1.067	4.251	H
ATOM	242	H07	LYS	A	12	1.484	-1.325	5.713	H
ATOM	243	H08	LYS	A	12	5.533	-0.391	2.229	H
ATOM	244	H09	LYS	A	12	5.475	1.024	4.964	H
ATOM	245	H10	LYS	A	12	3.662	-1.027	3.573	H
ATOM	246	H11	LYS	A	12	2.771	0.678	5.952	H
ATOM	247	H12	LYS	A	12	1.411	-1.187	4.027	H
ATOM	248	N	GLY	A	13	7.766	0.797	0.215	N
ATOM	249	CA	GLY	A	13	8.657	0.144	-0.727	C
ATOM	250	C	GLY	A	13	8.519	-1.380	-0.752	C
ATOM	251	O	GLY	A	13	9.481	-2.095	-1.064	O
ATOM	252	H	GLY	A	13	7.027	1.371	-0.183	H
ATOM	253	H01	GLY	A	13	9.704	0.341	-0.488	H
ATOM	254	H02	GLY	A	13	8.435	0.552	-1.716	H
ATOM	255	N	LYS	A	14	7.305	-1.893	-0.380	N
ATOM	256	CA	LYS	A	14	7.275	-3.317	-0.092	C
ATOM	257	C	LYS	A	14	6.099	-4.074	-0.686	C
ATOM	258	O	LYS	A	14	4.972	-3.616	-0.918	O
ATOM	259	CB	LYS	A	14	7.324	-3.552	1.448	C
ATOM	260	CG	LYS	A	14	8.697	-3.092	1.958	C
ATOM	261	CD	LYS	A	14	8.858	-3.085	3.495	C
ATOM	262	CE	LYS	A	14	10.067	-2.189	3.782	C
ATOM	263	NZ	LYS	A	14	10.467	-2.222	5.278	N
ATOM	264	H	LYS	A	14	6.491	-1.302	-0.199	H
ATOM	265	H01	LYS	A	14	9.684	-1.906	5.868	H
ATOM	266	H02	LYS	A	14	8.202	-3.696	-0.535	H
ATOM	267	H03	LYS	A	14	7.114	-4.600	1.679	H
ATOM	268	H04	LYS	A	14	8.846	-2.064	1.623	H
ATOM	269	H05	LYS	A	14	8.999	-4.101	3.879	H
ATOM	270	H06	LYS	A	14	9.822	-1.157	3.513	H
ATOM	271	H07	LYS	A	14	11.279	-1.609	5.451	H
ATOM	272	H08	LYS	A	14	6.530	-2.946	1.902	H
ATOM	273	H09	LYS	A	14	9.486	-3.709	1.513	H
ATOM	274	H10	LYS	A	14	7.961	-2.652	3.955	H
ATOM	275	H11	LYS	A	14	10.951	-2.523	3.234	H
ATOM	276	H12	LYS	A	14	10.719	-3.183	5.560	H
ATOM	277	N	LYS	A	15	6.423	-5.394	-0.885	N
ATOM	278	CA	LYS	A	15	5.413	-6.427	-1.014	C

```
ATOM    279  O   LYS A  15       4.998  -6.016   1.372                       O
ATOM    280  CB  LYS A  15       6.037  -7.780  -1.421                       C
ATOM    281  CG  LYS A  15       6.696  -7.762  -2.813                       C
ATOM    282  CD  LYS A  15       7.291  -9.157  -3.133                       C
ATOM    283  CE  LYS A  15       7.935  -9.168  -4.524                       C
ATOM    284  NZ  LYS A  15       8.522 -10.566  -4.841                       N
ATOM    285  H   LYS A  15       7.354  -5.700  -0.627                       H
ATOM    286  H01 LYS A  15       9.243 -10.824  -4.147                       H
ATOM    287  H02 LYS A  15       4.661  -6.132  -1.750                       H
ATOM    288  H04 LYS A  15       5.236  -8.524  -1.397                       H
ATOM    289  H05 LYS A  15       7.483  -7.002  -2.847                       H
ATOM    290  H06 LYS A  15       6.489  -9.904  -3.087                       H
ATOM    291  H07 LYS A  15       8.769  -8.467  -4.593                       H
ATOM    292  H08 LYS A  15       8.958 -10.581  -5.778                       H
ATOM    293  H09 LYS A  15       6.778  -8.068  -0.663                       H
ATOM    294  H10 LYS A  15       5.943  -7.501  -3.566                       H
ATOM    295  H11 LYS A  15       8.040  -9.409  -2.373                       H
ATOM    296  H12 LYS A  15       7.208  -8.961  -5.310                       H
ATOM    297  H13 LYS A  15       7.781 -11.285  -4.817                       H
TER     298      LYS A  15
HETATM  299  C           0       4.730  -6.603   0.340                       C
HETATM  300  O           0       3.756  -7.561   0.240                       O
HETATM  301  H           0       3.341  -7.737   1.121                       H
END
CONECT  278  299
CONECT  279  299
CONECT  299  278  279  300
CONECT  300  299  301
CONECT  301  300
```
